# Characterization of ‘*QTL-hotspot*’ introgression lines reveals physiological mechanisms and candidate genes associated with drought adaptation in chickpea

**DOI:** 10.1093/jxb/erac348

**Published:** 2022-08-25

**Authors:** Rutwik Barmukh, Manish Roorkiwal, Girish P Dixit, Prasad Bajaj, Jana Kholova, Millicent R Smith, Annapurna Chitikineni, Chellapilla Bharadwaj, Sheshshayee M Sreeman, Abhishek Rathore, Shailesh Tripathi, Mohammad Yasin, Adiveppa G Vijayakumar, Someswar Rao Sagurthi, Kadambot H M Siddique, Rajeev K Varshney

**Affiliations:** Centre of Excellence in Genomics and Systems Biology, International Crops Research Institute for the Semi-Arid Tropics (ICRISAT), Hyderabad, India; Department of Genetics, Osmania University, Hyderabad, India; Centre of Excellence in Genomics and Systems Biology, International Crops Research Institute for the Semi-Arid Tropics (ICRISAT), Hyderabad, India; The UWA Institute of Agriculture, The University of Western Australia, Perth, Western Australia, Australia; Khalifa Center for Genetic Engineering and Biotechnology, United Arab Emirates University, Al-Ain, United Arab Emirates; ICAR - Indian Institute of Pulses Research (IIPR), Kanpur, India; Centre of Excellence in Genomics and Systems Biology, International Crops Research Institute for the Semi-Arid Tropics (ICRISAT), Hyderabad, India; Crops Physiology & Modeling, International Crops Research Institute for the Semi-Arid Tropics (ICRISAT), Hyderabad, India; Department of Information Technologies, Faculty of Economics and Management, Czech University of Life Sciences Prague, Kamýcká 129, Prague, Czech Republic; Centre of Excellence in Genomics and Systems Biology, International Crops Research Institute for the Semi-Arid Tropics (ICRISAT), Hyderabad, India; Queensland Alliance for Agriculture and Food Innovation, The University of Queensland, Australia; Centre of Excellence in Genomics and Systems Biology, International Crops Research Institute for the Semi-Arid Tropics (ICRISAT), Hyderabad, India; The UWA Institute of Agriculture, The University of Western Australia, Perth, Western Australia, Australia; ICAR - Indian Agricultural Research Institute (IARI), Delhi, India; Department of Crop Physiology, University of Agricultural Sciences, Bengaluru, India; Centre of Excellence in Genomics and Systems Biology, International Crops Research Institute for the Semi-Arid Tropics (ICRISAT), Hyderabad, India; ICAR - Indian Agricultural Research Institute (IARI), Delhi, India; RAK College of Agriculture, Rajmata Vijayaraje Scindia Krishi Vishwa Vidyalaya, Gwalior, India; UAS-Dharwad Regional Agricultural Research Station, Vijayapura, India; Department of Genetics, Osmania University, Hyderabad, India; The UWA Institute of Agriculture, The University of Western Australia, Perth, Western Australia, Australia; Centre of Excellence in Genomics and Systems Biology, International Crops Research Institute for the Semi-Arid Tropics (ICRISAT), Hyderabad, India; The UWA Institute of Agriculture, The University of Western Australia, Perth, Western Australia, Australia; Centre for Crop and Food Innovation, State Agricultural Biotechnology Centre, Murdoch University, Murdoch, Western Australia, Australia; University of Birmingham, UK

**Keywords:** Chickpea, *Cicer arietinum*, drought stress, haplotypes, introgression lines, legume, marker-assisted backcrossing, transpiration efficiency, whole-genome sequencing

## Abstract

‘*QTL-hotspot*’ is a genomic region on linkage group 04 (CaLG04) in chickpea (*Cicer arietinum*) that harbours major-effect quantitative trait loci (QTLs) for multiple drought-adaptive traits, and it therefore represents a promising target for improving drought adaptation. To investigate the mechanisms underpinning the positive effects of ‘*QTL-hotspot*’ on seed yield under drought, we introgressed this region from the ICC 4958 genotype into five elite chickpea cultivars. The resulting introgression lines (ILs) and their parents were evaluated in multi-location field trials and semi-controlled conditions. The results showed that the ‘*QTL-hotspot*’ region improved seed yield under rainfed conditions by increasing seed weight, reducing the time to flowering, regulating traits related to canopy growth and early vigour, and enhancing transpiration efficiency. Whole-genome sequencing data analysis of the ILs and parents revealed four genes underlying the ‘*QTL-hotspot*’ region associated with drought adaptation. We validated diagnostic KASP markers closely linked to these genes using the ILs and their parents for future deployment in chickpea breeding programs. The *CaTIFY4b-H2* haplotype of a potential candidate gene *CaTIFY4b* was identified as the superior haplotype for 100-seed weight. The candidate genes and superior haplotypes identified in this study have the potential to serve as direct targets for genetic manipulation and selection for chickpea improvement.

## Teaser

Allelic variation in the ‘*QTL-hotspot*’ region confers drought adaptation in chickpea by influencing seed weight, flowering time, early vigour, and transpiration efficiency under water deficit.

## Introduction

Global food security is one of the most critical challenges confronting humanity today due to increasing environmental fluctuations. Rainfed agriculture covers about 80% of the total cropped area and contributes more than 60% of the world’s food productivity (UNCTAD, 2011). However, water availability in rainfed farming systems varies considerably, and this often results in short-term dry spells and/or long-term droughts. Improved crop varieties with better adaptation to water-limited environments are urgently needed to maintain and enhance yields, particularly in South Asia and sub-Saharan Africa (Varshney *et al*., 2021a, 2021b).

Chickpea (*Cicer arietinum*) is a major cool-season legume crop typically grown in the semi-arid tropics and it serves as a key source of livelihood for subsistence farming communities in developing countries. Global annual production of chickpea is ~15.08 million tonnes from ~14.84 million hectares, and India represents the highest chickpea producer with ~73% of the global production (FAOSTAT, 2020). Chickpea is cultivated on residual soil moisture in India during the post-rainy season, and it is often exposed to late-season water deficit towards the end of the crop cycle (Kashiwagi *et al.*, 2013; Hajjarpoor *et al.*, 2018). Chickpea production in rainfed systems is limited mainly by drought stress in the later stages of development (terminal drought), causing severe yield losses (64% on average in India; Hajjarpoor *et al.*, 2018). Breeding for improved root growth and architecture has been one of the major targets in the last three decades for increasing drought adaptation in chickpea (Varshney *et al.*, 2013a; Kashiwagi *et al.*, 2015). High root-length density has been proposed as the key drought avoidance trait contributing to chickpea seed yield under terminal drought conditions (Kashiwagi *et al.*, 2006a). While this is clearly important, it is also crucial to consider that it only applies as long as the roots are able to facilitate water uptake at critical growth stages for the plant. The water-use pattern is important for crops grown in regions with limited soil water availability because their reproductive success mainly depends on sustained water-use into the reproductive stage (Pang *et al.*, 2017). An important feature of water management is controlling the overall water loss at the canopy level through related growth traits such as plant vigour and leaf size (Kholová *et al.*, 2014; Vadez *et al.*, 2015; Sivasakthi *et al.*, 2018). At the plant level, high transpiration efficiency (TE)—which is related to increased biomass accumulation and/or reduced water transpiration—confers a conservative water-use pattern and it can be advantageous under terminal drought conditions (Vadez *et al.*, 2014). However, limited data exist for plant vigour, TE and their interactions for drought adaptation in chickpea (Nguyen *et al.*, 2021).

Marker-assisted backcrossing (MABC) has been used in many crops for introgressing beneficial quantitative trait loci (QTLs) into elite cultivars to develop introgression lines (ILs; Placido *et al.*, 2013; Varshney *et al*., 2013a, 2021c; Roorkiwal *et al.*, 2020). Varshney *et al.* (2014) identified a genomic region on linkage group 04 (CaLG04) of chickpea, termed ‘*QTL-hotspot*’, that harbours multiple QTLs for drought tolerance component traits and accounts for up to 58.2% of phenotypic variation explained. Relative to the recurrent parents, introgression of this ‘*QTL-hotspot*’ into elite Indian chickpea cultivars using MABC increases the seed yield of ILs by up to 16% under drought conditions and improves root traits such as total length, length density, surface area, and volume, among others (Varshney *et al.*, 2013a; Roorkiwal *et al.*, 2020; Bharadwaj *et al.*, 2021). Although this genomic region improves yield in water-limited environments, the underlying mechanisms remain largely unclear. Root growth parameters have been found to vary among ILs (Varshney *et al.*, 2013a; Bharadwaj *et al.*, 2021), but differences in water-uptake patterns and TE have not been examined. The identification of candidate gene(s) and diagnostic markers associated with key drought-adaptive traits will pave the way for the development of improved chickpea varieties that are adapted for future climates.

In the present study, we used drought-adaptive ILs developed by applying a MABC strategy to unravel the physiological and molecular bases of drought adaptations controlled by the ‘*QTL-hotspot*’ region in chickpea. The overall objectives were to identify whether the ILs differed in yield, phenology, and agronomic traits compared to their recurrent parents under field conditions; to clarify whether these ILs were associated with specific traits related to canopy growth; to assess the pattern of water-use and TE of the ILs under water deficit; and to identify the candidate gene(s) and superior haplotypes associated with key drought-adaptive traits in chickpea.

## Materials and methods

### Plant material

We used five elite cultivars of chickpea (*Cicer arietinum*) as recurrent parents, namely Pusa 372, ICCV 10, RSG 888, Pusa 362, and JG 11. Pusa 372 was developed and released for cultivation by ICAR-Indian Agricultural Research Institute (ICAR-IARI), New Delhi, in 1993 and has an average time to maturity of 135–150 d. ICCV 10 is a short- to mid-season maturing (95–100 d) and high-yielding variety developed at ICRISAT, India, in 1992. RSG 888 was developed by Dr Rajendra Prasad Central Agricultural University, India, in 2003 and is suitable for rainfed conditions with an average time to maturity of 130–135 d. Pusa 362 was developed at ICAR-IARI in 1995, and is a wilt-tolerant and bold-seeded variety that matures in 145–150 d. JG 11 was developed at Jawaharlal Nehru Krishi Vishwa Vidyalaya in collaboration with ICRISAT and released in 1999, and is an early-maturing variety (95–100 d). It is a bold-seeded variety, resistant to wilt and moderately resistant to dry root rot, and is most suitable for rainfed conditions in the southern zone of India. We used the drought-adapted cultivar ICC 4958 containing the ‘*QTL-hotspot*’ region as a donor parent. Drought adaptation of ICC 4958 was based on its yields under terminal drought stress over several years of field experiments and screening for root traits related to drought adaptation (Kashiwagi *et al.*, 2006a).

### Development of introgression lines using marker-assisted backcrossing

To introgress ‘*QTL-hotspot*’ into the background of the five elite cultivars, they were first crossed to ICC 4958, followed by 2–3 backcrosses with the respective recurrent parent to recover most of its genetic background. Based on marker polymorphism between the donor and recurrent parents, the presence or absence of the ‘*QTL-hotspot*’ region was evaluated at each backcross using simple sequence repeat (SSR) flanking markers (ICCM0249, NCPGR127, TAA170, NCPGR21, TR11, GA24, GA11, and STMS11) on CaLG04 (Varshney *et al.*, 2014). Background selection was made using highly polymorphic SSR markers distributed across eight linkage groups, as described previously (Bharadwaj *et al.*, 2021). Individual plants that were heterozygous at the ‘*QTL-hotspot*’ region (based on foreground selection) and had the highest recurrent parent genome recovery (based on background selection) were selected for further crossing. After backcrossing, two rounds of selfing were performed to develop superior ILs. Full details of the ILs and their recurrent parents used in this study are given in Table 1.

**Table 1.** The pedigree of the seven ‘*QTL-hotspot*’ introgression lines examined in this study.

Introgression line	Donor parent	Recurrent parent	Pedigree
BGM 10216	ICC 4958	Pusa 372	(Pusa 372×ICC 4958)×2×Pusa 372
DIBG 205	ICC 4958	ICCV 10	[(ICCV 10×ICC 4958)×3×ICCV 10] - 21
BGM 10218	ICC 4958	RSG 888	RSG 888×[RSG 888×(RSG 888×ICC 4958) - 4]
BG 4005	ICC 4958	Pusa 362	Pusa 362×[Pusa 362×(Pusa 362×ICC 4958) - 6]
BG 3097	ICC 4958	Pusa 362	[(Pusa 362×ICC 4958)×2×Pusa 362] - 51
DIBG 505	ICC 4958	JG 11	(JG 11×ICC 4958)×3×JG 11 - 3
RVSS 51	ICC 4958	JG 11	[(JG 11×ICC 4958)×3×JG 11] - 34

### Multi-location field evaluations

The ILs derived from the marker-assisted backcrossing (BC_2_F_3_ or BC_3_F_3_ progenies) and their recurrent parents were screened under rainfed field conditions at up to eight locations across India during the 2018–19 post-rainy season. Phenotypic data for some lines at some locations were not available due to limitations in the available resources and/or environmental factors. The genotypes were evaluated for yield and agro-morphological traits in Advanced Varietal Trials 2 (AVT2) of the ICAR-All India Coordinated Research Project (AICRP) on Chickpea in the central and southern zones of India at Gulbarga (17.3297°N, 76.8343°E), Coimbatore (11.0168°N, 76.9558°E), Vijayapur (16.8302°N, 75.7100°E), Badnapur (19.8682°N, 75.7256°E), Rahuri (19.3951°N, 74.6521°E), Nandyal (15.4777°N, 78.4873°E), Arnej (22.5825°N, 72.2667°E), and Sehore (23.2032°N, 77.0844°E). The field trials were conducted in a randomized complete block design with four replications and followed the recommended agronomic practices for each genotype. Seeds were sown at 80 kg ha^–1^ with 30 cm between rows, and thinned at the seedling stage to 10 cm between plants within rows, which resulted in a final density of 33 plants m^–2^. Optimal doses of fertilizer were applied during the trial (20 kg N + 40 kg P_2_O_5_ + 20 kg S + 25 kg ZnSO_4_ ha^–1^). Plants were harvested at maturity, and the following agro-morphological traits were assessed: yield ha^–1^, 100-seed weight, plant height, days to 50% flowering, and days to maturity. Data for soil moisture content (%) and total rainfall (mm) were recorded at 30-d intervals throughout the growing season from sowing until maturity.

### Evaluation of traits related to canopy growth using the LeasyScan platform

A high-throughput phenotyping platform, LeasyScan, based on a novel 3D imaging technique was used to evaluate the continuous progress of canopy growth traits in the initial stages of plant development (http://gems.icrisat.org/leasyscan/; Vadez *et al.*, 2015). ILs and their parents were grown under fully irrigated conditions in an experiment conducted during the post-rainy season (December 2018 to January 2019) at ICRISAT, Patancheru, India. Trays of 65 × 40 cm and depth of 100 cm were filled with Vertisol collected at the ICRISAT site and placed in the LeasyScan platform. Twelve seeds were sown per tray and thinned to eight seedlings at 14 d after sowing (DAS), resulting in a final density of 32 plants m^–2^, equivalent to that in the field. The mean day/night temperatures during crop growth were 31.0/5.2 °C and the corresponding relative humidities were 26/99%. The trays were irrigated regularly to maintain them in a well-watered state. Each tray was imaged using high-throughput PlantEye scanners (Phenospex). The following traits related to canopy growth and biomass were assessed, as described previously (Sivasakthi *et al.*, 2018): 3D-leaf area, 3D-leaf area growth rate, projected leaf area, leaf area index, plant height, specific leaf area, digital biomass, digital biomass growth rate, shoot dry weight, and specific leaf weight, where digital biomass was calculated as the product of height and 3D leaf area. The evaluated traits are listed in Supplementary Table S1. The genotypes were visually scored for plant vigour on a scale of 1 to 6, where 1 is late vigour and 6 is early vigour (Sivasakthi *et al.*, 2018). Plant growth rates (3D-leaf area growth rate and digital biomass growth rate) were estimated based on the mean differences between consecutive days during the exponential growth stage.

### Evaluation of plant growth and responses to drought stress in a pot experiment

Plants were grown in pots (20 cm depth × 25 cm diameter) from July–September 2019 in a glasshouse at ICRISAT, Patancheru, India, under near-optimal conditions (mean day/night temperature 32/25 °C, relative humidity 40–80% during the day). Two plants were maintained per pot. A 3:1 mix of sand:soil was used, with the Vertisol soil collected from ICRISAT, Patancheru. The soil was fertilized with di-ammonium phosphate at 100 mg kg^–1^ soil.

The transpiration response of plants to progressive exposure to drought stress was evaluated in the ILs and their recurrent and donor parents. The experiment was designed to assess the relationship between traits related to water-use and the known performance of the ILs in the field with the putative presence/absence of an introgressed ‘*QTL-hotspot*’ region. Plants were divided between a well-watered (WW) and a water stress (WS) treatment. The treatments were imposed during the vegetative stage, starting at 28 DAS with the pots completely saturated with water and allowed to drain overnight. The next morning, the top of the soil was covered with a round plastic sheet and a thin layer of low-density polyethylene granules in order to prevent soil evaporation. The pots were weighed every 3 d from 29 DAS to 44 DAS, then every 6 d to 56 DAS. The WW treatment was maintained at ~80% field capacity for the duration of the experiment. The WS treatment was subjected to progressive drought stress by only partially compensating for water loss from transpiration: the plants were permitted to lose no more than 150 g of water every 3 d during the reproductive and pod-filling stages. Any excess transpiration over to this maximum was added back to the pots by watering (Kholová *et al.*, 2009). For evaluation at the reproductive stage, plants were harvested at 42 DAS. For evaluation at the pod-filling stage, plants were harvested when the transpiration of the WS plants was ~10–20% of that of the WW plants. Plants from three replicate pots were sampled each time. Shoot fresh weight, root fresh weight, and total fresh weight of the plants were measured on a per-pot basis. For instance, the shoot fresh weight of both plants in a pot was considered as a single data point for the pot. The TE was calculated by dividing the total shoot fresh weight by the total water transpired.

### Carbon isotope discrimination

At 37 DAS, two leaflets were sampled from each tray of the plants grown using the LeasyScan platform by detaching with a razor blade. The leaflets selected were fully expanded, non-shaded leaflets of the primary branches. Both surfaces of each leaflet were gently wiped upon collection with tissue to remove leaf acids. Samples were oven-dried at 65 °C and ground using an oscillating matrix mill. Carbon isotope discrimination of the ground leaf material was determined using an isotope ratio mass spectrometer (ThermoFisher Scientific) at the Centre for Carbon, Water and Food, The University of Sydney, Australia. Carbon isotope discrimination (∆^13^C) was calculated by comparing the ratio of ^13^C to ^12^C against the standard Peedee Belemnite (PDB). Carbon isotope discrimination expressed as ‰ was calculated assuming ^13^C of the air of –8.0‰ (Hall *et al.*, 1994).

### DNA extraction, whole-genome sequencing, and SNP identification

Genomic DNA was extracted from the leaves of 15-day-old seedlings using a high-throughput mini-DNA extraction method as described by Jain *et al.* (2019). The DNA quality was checked on 0.8% agarose gel. The DNA quantity was assessed using a Qubit^®^ 2.0 fluorometer (Life Technologies, ThermoFisher Scientific). High-quality DNA was used for sequencing.

Sequencing of seven ILs and four recurrent parental lines was performed on a HiSeq^TM^ 2500 using a TruSeq DNA Sample Prep Kit LT (set A) FC-121-2001 (both Illumina) following the manufacturer’s protocol. In addition, sequencing data generated for two genotypes (ICC 4958 and ICCV 10) in earlier studies (Thudi *et al*., 2016a, 2016b) was also used in the present study. Due to the low quality of sequencing data obtained for the JG 11 genotype, we excluded it and its ILs (DIBG 505 and RVSS 51) from further analysis.

Adaptor sequences and low-quality reads were trimmed from the raw data of the remaining 10 genotypes using Trimmomatic v0.39 (http://www.usadellab.org/cms/?page=trimmomatic). BWA mem v0.7.17 (http://bio-bwa.sourceforge.net/) was used to align the clean reads to the chickpea reference genome (Varshney *et al.*, 2013b). The alignment files generated were then used for variant discovery using GATK v4.1.8 (https://gatk.broadinstitute.org/hc/en-us), according to the GATK best practices. Single-nucleotide polymorphisms (SNPs) were extracted and compared within the ~300-kb ‘*QTL-hotspot*’ region on pseudomolecule Ca4 in chickpea. SNPs with the same alleles across both parents and the corresponding IL were removed from further analysis. Candidate genes present within the ‘*QTL-hotspot*’ region were retrieved from the chickpea draft genome (CaGAv1.0) (Varshney *et al.*, 2013b) and were searched against the NCBI-nr protein database via the BLAST program. The gene ontology terms related to the genes were searched using the BLAST2GO software (Conesa *et al.*, 2005).

### Validation of KASP assay markers

Nine SNPs were selected and converted into Kompetitive Allele Specific PCR (KASP) markers to validate the polymorphic SNPs obtained within the ~300-kb ‘*QTL-hotspot*’ region using the whole-genome sequencing data. The developed markers were designated as chickpea KASP assay markers (CKAMs) (Hiremath *et al.*, 2012). All nine CKAMs were used to validate the 12 genotypes, namely the six ILs, five recurrent parents, and the one donor parent (ICC 4958).

### Haplotype analysis

The haplotypes for the *CaTIFY4b* gene identified from a SNP set for 1548 desi chickpea accessions generated in our previous study (Varshney *et al.*, 2021d) were used for further analysis: all the donor and recurrent parents used in the present study were desi types, and hence only these accessions were selected for haplotype analysis. Data for 100-seed weight for the 1548 accessions measured at ICRISAT during the 2014–15 cropping season (Varshney *et al.*, 2021d) were used for haplo-pheno analysis. The superior haplotype was identified by comparing the accessions classified based on haplotype groups with the 100-seed weight data (Sinha *et al.*, 2020). Duncan’s multiple range test was performed to identify the phenotypic performance of each haplotype.

### Statistical analyses

ANOVA was computed using the SAS software (SAS Institute Inc.). Differences among genotype means were analysed using Tukey’s test (at *P*<0.05) whilst Student’s *t*-test was used to analyse pairs of means, using the statistical software GenStat version 15 (VSN International, UK). Broad-sense heritability (*h*^2^) for individual locations and across locations was computed for all traits using the R software. The formula used was *h*^2^=σ^2^_g_/σ^2^_p_, where σ^2^_p_= σ^2^_g_+(σ^2^_*ε*_/*r*) for individual locations and σ^2^_p_= σ^2^_g_+(σ^2^_g×e_/*l*)+(σ^2^_*ε*_ /*lr*) for combined locations, where σ^2^_g_, σ^2^_g×e_, and σ^2^_*ε*_ represent the genotype, genotype × environment, and residual variances, respectively, *l* is the number of locations, and *r* is the number of replications. Principal component analysis (PCA) factor graphs were generated using the ‘FactoMineR’ package in the R statistical computing environment.

## Results

### ‘*QTL-hotspot*’ improves yield performance under rainfed field conditions

**Fig. 1.**
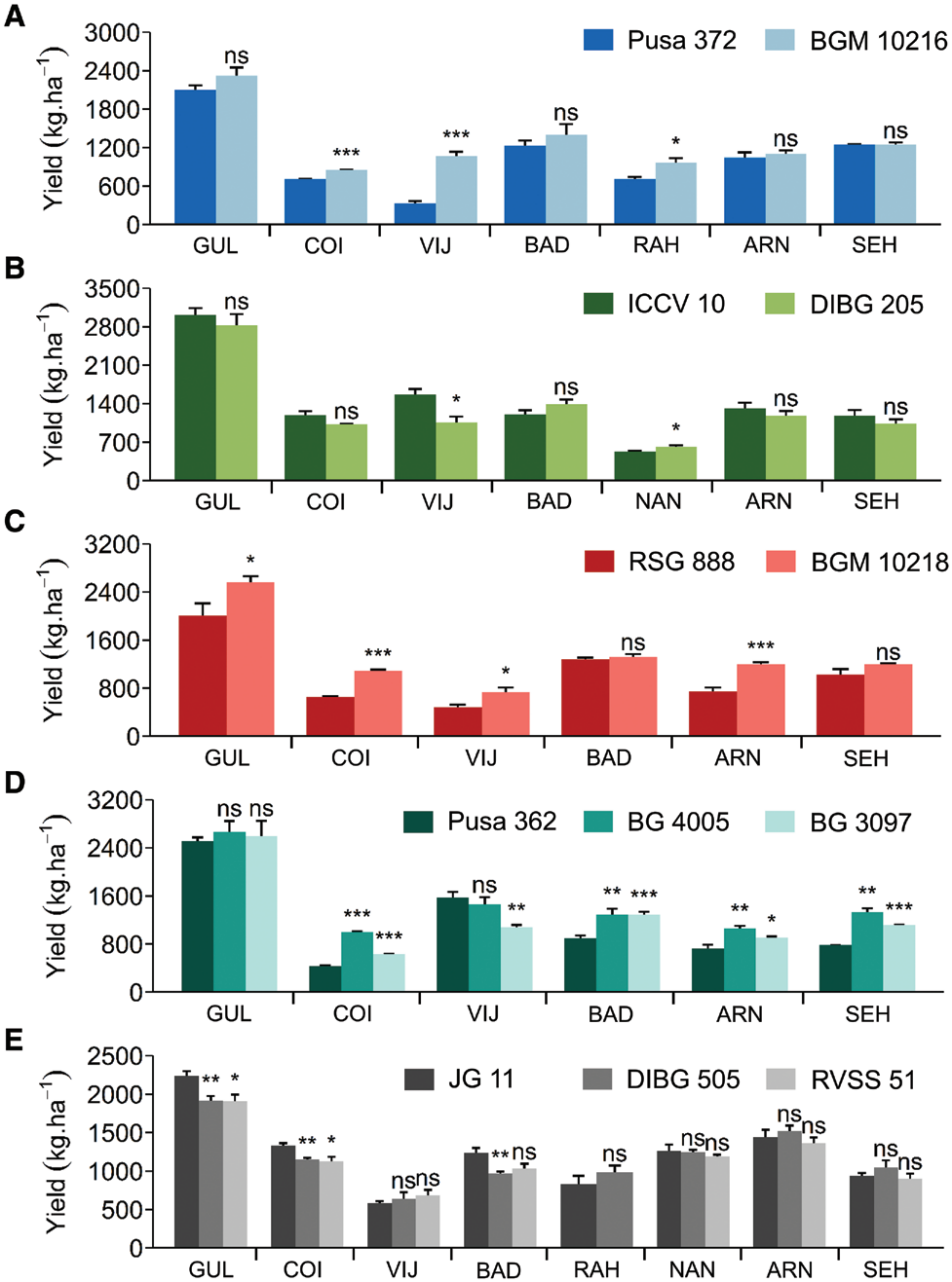
Yield performance of chickpea introgression lines (ILs) and their recurrent parental lines in the rainfed multi-location field trials in 2018–2019. The first-named line is the recurrent parent and details of the lines are given in Table 1. The donor parent in each case was ICC 4958, which possess ‘*QTL-hotspot*’. Mean plot yields are shown for (A) Pusa 372 and BGM 10216, (B) ICCV 10 and DIBG 205, (C) RSG 888 and BGM 10218, (D) Pusa 362, BG 4005, and BG 3097, (E) JG 11, DIBG 505, and RVSS 51. Data are means (±SE), *n*=4. Significant differences between the ILs and their recurrent parent were determined using Student’s *t*-test: *P<0.05, **P<0.01, ***P<0.001; ns, not significant. The locations are as follows: GUL, Gulbarga; COI, Coimbatore; VIJ, Vijayapur; BAD, Badnapur; RAH, Rahuri; NAN, Nandyal; ARN, Arnej; SEH, Sehore. Results for some lines at some locations were not available due to limitations in the available resources and/or environmental factors.

The ‘*QTL-hotspot*’ region was introgressed into five elite chickpea cultivars using the MABC method (Supplementary Fig. S1; Table 1) to investigate its influence on yield performance. We assessed the seed yield of seven ILs under rainfed field conditions at up to eight locations across India (Supplementary Table S1). We found that the ILs with genetic backgrounds of Pusa 372, RSG 888, and Pusa 362 had an overall mean yield advantage of up to 30.7% over their recurrent parents. For example, the seed yield of BGM 10216 was significantly increased by 19.9–221.6% over the recurrent parent Pusa 372 at three of the seven locations evaluated (Fig. 1A), with an overall mean yield advantage of 21.4% (yields ranged from 846–2320 kg ha^–1^ in BGM 10216 and 331–2101 kg ha^–1^ in Pusa 372). Seed yield did not significantly differ between DIBG 205 and the recurrent parent ICCV 10 in five of seven locations (Fig. 1B). BGM 10218 outperformed the recurrent parent RSG 888 (by 3.1–66.9%) in four of six locations evaluated (Fig. 1C), with an overall mean yield advantage of 30.7%. Seed yields of BG 4005 and BG 3097 significantly increased by up to 130.9% and 46.5%, respectively, over the recurrent parent Pusa 362, at four of six locations for both genotypes (Fig. 1D), with an overall mean yield advantage of 27.4% and 10.3%, respectively. In contrast, seed yield of DIBG 505 and RVSS 51 significantly decreased by up to 21.4% and 16.7% compared with the recurrent parent JG 11 at three and two locations, respectively (Fig. 1E). Substantial genotypic variability and high broad-sense heritability (*h*^2^=71–99%) was observed for seed yield at the various locations evaluated in the rainfed field experiment (Supplementary Table S2). The ANOVA for seed yield indicated a significant genotype × environment (G×E) effect (*P*<0.001; Supplementary Table S3).

The ‘which-won-where’ view of the genotype and G×E (GGE) principal component bi-plot was used to determine the best-performing genotype in each environment and each ‘mega-environment’ (Supplementary Fig. S2A). First, the genotypes furthest from the origin were connected to form a polygon containing all the genotypes. ICCV 10, JG 11, DIBG 505, RSG 888, and Pusa 362 were positioned at the vertices of the polygon, representing the best- or worst-performing genotypes in some or all environments. Next, the bi-plot was divided into five sectors, revealing two mega environments, one of which comprised the locations Gulbarga, Vijayapur, Badnapur, and Sehore, whilst the other comprised Coimbatore, Arnej, Rahuri, and Nandyal. The genotype in each sector furthest from the bi-plot origin was the best-performing one for that environment, while those located closer to the origin were less sensitive to changing environments. Accordingly, the best-yielding genotypes were ICCV 10 in the first mega-environment, and JG 11 and DIBG 505 in the second mega-environment. BGM 10216 was closest to the bi-plot origin, representing the most stable genotype across all environments (Supplementary Fig. S2A, B). Data for soil moisture content and total rainfall during the cropping season at the different locations were recorded at 30-d intervals (Supplementary Tables S4, S5). Notably, the soil moisture contents during the early (60–90 DAS) and late (90–120 DAS) pod-filling stages were found to differentiate the two mega-environments for seed yield. Thus the soil moisture content was higher in the first mega-environment than in the second during both the early pod-filling stage (20.9–40.0% versus 12.0–16.6%) and the late pod-filling stage (18.0-19.8% versus 14.8%).

### ‘*QTL-hotspot*’ increases individual seed weight and reduces the time to flowering

We evaluated the yield-component trait 100-seed weight of the ILs over their recurrent parents under rainfed field conditions (Supplementary Table S1). The pooled mean data from all locations indicated that 100-seed weight markedly increased (by 38.7–53.1%) in most ILs compared to their recurrent parents, except for BG 4005 and BG 3097 (Table 2). BGM 10218 increased the most (53.1%) compared to the recurrent parent RSG 888, and BG 3097 increased the least (0.4%) compared to the recurrent parent Pusa 362. In terms of individual locations, 100-seed weight significantly increased in all the ILs over their recurrent parents, except those in the background of Pusa 362, at a minimum of five of the six-to-eight locations evaluated. The 100-seed weight varied significantly among genotypes (*P*<0.001) and had high broad-sense heritability (*h*^2^=64–99%) across locations in the rainfed field experiment (Supplementary Table S2). ANOVA revealed highly significant effects (*P*<0.001) of genotype, environment, and their interaction on 100-seed weight (Supplementary Table S3), suggesting that the presence/absence of the ‘*QTL-hotspot*’ region differentiated between genotypes with high and low seed weight.

**Table 2.** Yield components, canopy growth, and phenology traits of the introgression lines and their recurrent parents evaluated in the rainfed multi-location field trials.

		Location								
Trait	Line^†^	Gulbarga	Coimbatore	Vijayapur	Badnapur	Rahuri	Nandyal	Arnej	Sehore	Pooled mean
100-seed weight	Pusa 372 (RP)	12.35 ± 0.48	14.07 ± 0.05	27.88 ± 1.46	12.40 ± 0.22	14.40 ± 0.26	–	13.30 ± 0.24	13.32 ± 0.24	15.39 ± 2.10
	BGM 10216	21.80 ± 0.50***	21.20 ± 0.27***	25.88 ± 1.60 ns	22.10 ± 0.39***	21.40 ± 0.38***	–	20.90 ± 0.37***	20.34 ± 0.36***	21.95 ± 0.69*
	ICCV 10 (RP)	17.50 ± 0.53	28.00 ± 0.16	20.92 ± 1.10	18.70 ± 0.33	–	23.10 ± 0.41	16.60 ± 0.30	16.54 ± 0.29	20.19 ± 1.59
	DIBG 205	27.95 ± 0.26***	28.82 ± 0.12**	25.62 ± 1.57*	32.10 ± 0.57***	–	28.30 ± 0.50***	31.00 ± 0.55***	26.95 ± 0.48***	28.68 ± 0.85***
	RSG 888 (RP)	13.50 ± 0.42	14.90 ± 0.20	25.30 ± 1.50	13.30 ± 0.24	–	–	14.70 ± 0.26	13.56 ± 0.24	15.88 ± 1.90
	BGM 10218	23.35 ± 0.30***	24.50 ± 0.29***	28.05 ± 1.64 ns	24.80 ± 0.44***	–	–	23.80 ± 0.42***	21.40 ± 0.38***	24.32 ± 0.89**
	Pusa 362 (RP)	22.65 ± 0.38	28.25 ± 0.26	26.65 ± 1.32	25.30 ± 0.45	–	–	26.00 ± 0.46	22.88 ± 0.41	25.29 ± 0.89
	BG 4005	23.80 ± 0.68 ns	33.95 ± 0.05***	24.53 ± 1.57 ns	25.00 ± 0.44 ns	–	–	25.90 ± 0.46 ns	23.38 ± 0.42 ns	26.09 ± 1.61 ns
	BG 3097	25.00 ± 0.66*	26.97 ± 0.06*	25.10 ± 1.89 ns	25.40 ± 0.45 ns	–	–	27.00 ± 0.48 ns	22.79 ± 0.41 ns	25.38 ± 0.63 ns
	JG 11 (RP)	20.60 ± 0.32	27.75 ± 0.25	28.73 ± 1.31	24.50 ± 0.44	24.20 ± 0.43	22.10 ± 0.39	24.50 ± 0.44	18.13 ± 0.32	23.81 ± 1.24
	DIBG 505	30.10 ± 0.61***	41.88 ± 0.13***	28.30 ± 0.77 ns	33.10 ± 0.59***	39.00 ± 0.69***	34.30 ± 0.61***	34.50 ± 0.61***	28.99 ± 0.52***	33.77 ± 1.69***
	RVSS 51	30.75 ± 0.85***	38.50 ± 0.29***	29.20 ± 1.59 ns	38.00 ± 0.68***	–	33.30 ± 0.59***	35.90 ± 0.64***	25.51 ± 0.45***	33.02 ± 1.82***
Plant height	Pusa 372 (RP)	37.00 ± 0.66	29.22 ± 0.30	37.55 ± 0.97	40.63 ± 0.93	30.30 ± 0.54	–	44.10 ± 0.78	40.40 ± 0.72	37.03 ± 2.08
	BGM 10216	37.00 ± 0.66 ns	31.20 ± 0.24**	34.60 ± 1.28 ns	36.37 ± 1.03*	30.10 ± 0.54 ns	–	41.50 ± 0.74 ns	40.40 ± 0.72 ns	35.88 ± 1.62 ns
	ICCV 10 (RP)	36.00 ± 0.64	31.18 ± 0.10	33.00 ± 1.26	42.43 ± 0.39	–	26.30 ± 0.47	42.10 ± 0.75	41.40 ± 0.74	36.06 ± 2.36
	DIBG 205	42.00 ± 0.75***	30.30 ± 0.47 ns	38.65 ± 1.73*	36.00 ± 1.66*	–	30.80 ± 0.55***	43.00 ± 0.77 ns	49.40 ± 0.88***	38.59 ± 2.60 ns
	RSG 888 (RP)	36.00 ± 0.64	27.88 ± 0.42	34.10 ± 1.79	40.57 ± 0.71	–	–	41.00 ± 0.73	44.60 ± 0.79	37.36 ± 2.44
	BGM 10218	38.00 ± 0.68 ns	31.25 ± 0.39**	33.95 ± 1.16 ns	42.23 ± 1.00 ns	–	–	41.70 ± 0.74 ns	41.00 ± 0.73*	38.02 ± 1.85 ns
	Pusa 362 (RP)	41.00 ± 0.73	30.80 ± 0.12	36.40 ± 2.06	37.53 ± 0.90	–	–	43.90 ± 0.78	42.80 ± 0.76	38.74 ± 1.99
	BG 4005	36.00 ± 0.64**	29.57 ± 0.34*	31.20 ± 1.38 ns	38.53 ± 0.29 ns	–	–	41.80 ± 0.74 ns	38.80 ± 0.69**	35.98 ± 1.93 ns
	BG 3097	44.00 ± 0.78*	30.95 ± 0.24 ns	35.90 ± 1.79 ns	37.70 ± 1.01 ns	–	–	44.00 ± 0.78 ns	40.60 ± 0.72 ns	38.86 ± 2.07 ns
	JG 11 (RP)	32.00 ± 0.57	29.65 ± 0.20	30.20 ± 0.78	34.73 ± 1.78	31.50 ± 0.56	28.00 ± 0.50	39.80 ± 0.71	31.20 ± 0.56	32.13 ± 1.29
	DIBG 505	40.00 ± 0.71***	29.43 ± 0.45 ns	32.50 ± 1.86 ns	42.90 ± 0.68**	30.10 ± 0.54 ns	30.30 ± 0.54*	42.20 ± 0.75 ns	40.80 ± 0.73***	36.03 ± 2.10 ns
	RVSS 51	43.00 ± 0.77***	32.70 ± 0.33***	34.00 ± 2.78 ns	35.23 ± 1.32 ns	–	33.50 ± 0.60***	43.20 ± 0.77*	39.00 ± 0.69***	37.23 ± 1.70*
Days to 50% flowering	Pusa 372 (RP)	44.25 ± 1.18	61.50 ± 0.65	62.00 ± 2.38	53.00 ± 0.41	56.00 ± 0.71	–	71.00 ± 1.08	70.00 ± 0.41	59.68 ± 3.58
	BGM 10216	47.25 ± 0.75 ns	60.75 ± 0.48 ns	53.75 ± 2.25*	54.00 ± 0.41 ns	50.25 ± 0.75**	–	63.75 ± 0.75**	66.33 ± 0.24***	56.58 ± 2.70 ns
	ICCV 10 (RP)	44.00 ± 1.41	45.75 ± 0.48	62.50 ± 2.90	47.00 ± 0.41	–	55.25 ± 0.75	64.25 ± 0.75	68.00 ± 0.41	55.25 ± 3.72
	DIBG 205	45.75 ± 1.03 ns	52.25 ± 0.48***	52.33 ± 2.19*	52.00 ± 0.00***	–	51.25 ± 0.63**	64.25 ± 0.63 ns	67.00 ± 0.41 ns	54.98 ± 2.90 ns
	RSG 888 (RP)	43.00 ± 1.00	62.75 ± 0.48	55.67 ± 2.33	51.67 ± 0.24	–	–	74.75 ± 0.85	69.00 ± 0.41	59.47 ± 4.77
	BGM 10218	43.75 ± 0.48 ns	52.75 ± 0.25***	60.00 ± 2.65 ns	48.33 ± 0.24***	–	–	59.50 ± 0.65***	59.00 ± 0.41***	53.89 ± 2.77 ns
	Pusa 362 (RP)	47.75 ± 0.85	46.75 ± 0.48	64.75 ± 1.25	51.00 ± 0.41	–	–	69.00 ± 1.22	68.00 ± 0.41	57.88 ± 4.27
	BG 4005	50.75 ± 0.75*	50.75 ± 0.48**	51.50 ± 0.65***	50.00 ± 0.41 ns	–	–	60.50 ± 0.29**	65.00 ± 0.41**	54.75 ± 2.60 ns
	BG 3097	45.75 ± 1.03 ns	49.50 ± 0.50**	60.25 ± 2.43 ns	51.00 ± 0.41 ns	–	–	68.00 ± 0.58 ns	62.33 ± 0.24***	56.14 ± 3.53 ns
	JG 11 (RP)	38.50 ± 0.50	49.50 ± 0.50	42.25 ± 1.75	52.33 ± 0.24	42.00 ± 0.71	36.50 ± 0.29	54.00 ± 0.71	57.33 ± 0.24	46.55 ± 2.74
	DIBG 505	38.00 ± 0.58 ns	50.25 ± 0.48 ns	41.00 ± 1.08 ns	54.33 ± 0.24***	37.25 ± 0.63**	37.25 ± 0.25 ns	51.00 ± 0.41*	56.67 ± 0.24 ns	45.72 ± 2.89 ns
	RVSS 51	39.75 ± 0.25 ns	54.75 ± 0.48***	40.00 ± 3.00 ns	55.00 ± 0.41**	–	36.50 ± 0.29 ns	49.75 ± 0.48**	57.00 ± 0.41 ns	47.54 ± 3.24 ns
Days to maturity	Pusa 372 (RP)	111.50 ± 0.96	97.50 ± 0.29	99.25 ± 0.48	108.00 ± 0.41	105.80 ± 0.48	–	128.20 ± 0.63	113.20 ± 0.48	109.10 ± 3.88
	BGM 10216	113.00 ± 0.71 ns	97.50 ± 0.29 ns	94.00 ± 0.41***	114.00 ± 0.41***	104.50 ± 0.29 ns	–	121.80 ± 0.48***	114.20 ± 0.48 ns	108.40 ± 3.80 ns
	ICCV 10 (RP)	115.00 ± 0.71	85.75 ± 0.48	99.25 ± 0.48	111.00 ± 0.41	–	118.20 ± 0.48	123.20 ± 0.63	113.20 ± 0.48	109.40 ± 4.83
	DIBG 205	112.00 ± 1.15 ns	90.75 ± 0.48***	99.50 ± 0.65 ns	111.00 ± 0.41 ns	–	112.80 ± 0.48***	125.80 ± 0.48*	119.00 ± 0.41***	110.10 ± 4.43 ns
	RSG 888 (RP)	104.50 ± 1.26	100.00 ± 0.41	103.80 ± 0.63	113.00 ± 0.41	–	–	126.50 ± 0.65	119.20 ± 0.48	111.20 ± 4.19
	BGM 10218	107.00 ± 0.71 ns	93.25 ± 0.48***	99.80 ± 0.85**	111.00 ± 0.41*	–	–	111.00 ± 0.71***	111.20 ± 0.48***	105.50 ± 3.04 ns
	Pusa 362 (RP)	114.50 ± 1.44	83.50 ± 0.29	99.25 ± 0.48	110.00 ± 0.41	–	–	131.50 ± 0.29	120.00 ± 0.41	109.80 ± 6.83
	BG 4005	114.50 ± 1.44 ns	91.50 ± 0.65***	97.67 ± 0.33 ns	108.30 ± 0.24*	–	–	127.50 ± 0.29***	109.00 ± 0.41***	108.10 ± 5.17 ns
	BG 3097	114.50 ± 1.19 ns	86.00 ± 0.41**	104.20 ± 0.63***	113.30 ± 0.24***	–	–	132.50 ± 0.29 ns	112.00 ± 0.41***	110.40 ± 6.19 ns
	JG 11 (RP)	112.20 ± 1.03	87.25 ± 0.63	95.00 ± 0.41	107.70 ± 0.24	105.00 ± 0.41	98.00 ± 0.00	103.20 ± 0.63	110.20 ± 0.48	102.30 ± 2.98
	DIBG 505	104.80 ± 0.75**	85.00 ± 0.41*	86.00 ± 0.41***	107.00 ± 0.41 ns	93.00 ± 0.41***	96.00 ± 0.41**	102.00 ± 0.41 ns	109.20 ± 0.48 ns	97.90 ± 3.30 ns
	RVSS 51	105.80 ± 1.25**	91.75 ± 0.48**	94.00 ± 0.41 ns	106.30 ± 0.24**	–	94.50 ± 0.50***	98.00 ± 0.82**	109.00 ± 0.41 ns	99.90 ± 2.64 ns

RP, recurrent parent. The ‘*QTL-hotspot*’ donor parent was ICC 4958. Details of the lines are given in Table 1.

Data are means (±SE), *n*=4. Phenotypic data for some lines at some locations were not available due to limitations in the available resources and/or environmental factors. Significant differences compared with the recurrent parent were determined using Student’s *t*-test: *P<0.05, **P<0.01, ***P<0.001; ns, not significant.

We also determined the variation in phenology traits (days to 50% flowering and days to maturity) of the ILs compared to their recurrent parents under rainfed field conditions (Supplementary Table S1). All the ILs except for RVSS 51 showed earlier flowering (up to 10.8%) relative to their recurrent parents for the pooled mean data (Table 2). BGM 10216 had significantly earlier flowering (up to 13.3%) over the recurrent parent Pusa 372 at four locations. Similarly, BGM 10218 had substantially earlier flowering (up to 20.1%) compared to the recurrent parent RSG 888 at four locations. Except for DIBG 205 and BG 3097, the ILs had decreased days to maturity for the pooled means across the locations (by 0.6–5.1%) compared to their recurrent parents. BGM 10218 had significantly decreased days to maturity (up to 12.3%) compared to the recurrent parent RSG 888 at five locations. In contrast, days to maturity significantly increased (up to 5.8%) in DIBG 205 compared to recurrent parent ICCV 10 at three locations. Both days to 50% flowering and days to maturity had significant genotypic variability (*P*<0.001) and high *h*^2^ (up to 99%) across locations in the rainfed field experiment (Supplementary Table S2). Moreover, days to 50% flowering had a significant G×E interaction effect (*P*<0.001; Supplementary Table S3).

### ‘*QTL-hotspot*’ controls canopy growth and biomass traits

**Fig. 2.**
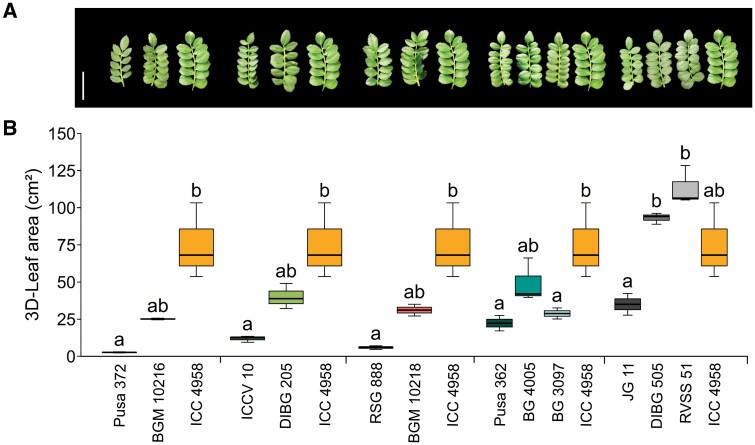
Phenotypic characterization of 3D-leaf area in chickpea introgression lines (ILs) and their parental lines using the LeasyScan platform. Plants were grown under well-watered conditions using the LeasyScan platform. Details of the lines are given in Table 1. The first-named line within each group is the recurrent parent, followed by the IL(s), and then the donor of ‘*QTL-hotspot*’, ICC 4958. (A) Representative images of leaves of each of the lines during the vegetative stage. The images correspond to the lines as labelled in the graph below. The scale bar is 3 cm. (B) Box-plots of the 3D-leaf area in the ILs and their parents during the vegetative stage. The boxes denote the 25th and 75th percentiles, whiskers denote the full data range, and the center lines denote the medians. Different letters indicate significant differences among genotypes as determined using ANOVA followed by Tukey’s test (*P<*0.05).

We evaluated traits related to canopy growth for the ILs and their parental lines to explore whether the control of water loss under non-limiting conditions at the vegetative stage was associated with drought adaptation in chickpea (Supplementary Table S1). DIBG 505 and RVSS 51 had significantly increased 3D-leaf area (165.9–223.9%), projected leaf area (162.3–220.3%), leaf area index (157.1–214.3%), and plant height (57.8–60.8%) over the recurrent parent JG 11 (Supplementary Table S6; Fig. 2). BGM 10216 and DIBG 205 had markedly increased plant height over the recurrent parents Pusa 372 (61.2%) and ICCV 10 (67.6%), respectively. Similar results but to a lesser extent occurred under rainfed conditions, with DIBG 205 having significantly increased plant height (by 2.1–19.3%) compared to the recurrent parent ICCV 10 at four locations (Table 2). DIBG 505 and RVSS 51 had significantly increased plant height over the recurrent parent JG 11 at four and five locations, respectively. In contrast, BG 4005 had decreased plant height (by 4.0–14.3%) compared to the recurrent parent Pusa 362 at three locations (Table 2). Apart from BG 4005 and BG 3097, all the ILs had significantly increased plant vigour compared to their respective recurrent parents under non-limiting water conditions (Supplementary Table S6). A time-course analysis of leaf area development suggested that all the ILs, except those with the genetic background of Pusa 362, rapidly increased their 3D-leaf area relatively early in the crop cycle compared to their recurrent parents (Supplementary Fig. S3). This was consistent with a marked increase in the 3D-leaf area growth rate for DIBG 505 and RVSS 51 compared to the recurrent parent JG 11 (Supplementary Table S6).

The biomass traits of the ILs and their parental lines were evaluated using the LeasyScan platform and in the pot experiment. Except for BG 4005 and BG 3097, all the ILs showed increased digital biomass compared to their recurrent parents under non-limiting water conditions (Supplementary Table S6), particularly for DIBG 505 (257.5%) and RVSS 51 (336.0%) and their recurrent parent JG 11. The digital biomass growth rate also significantly increased for DIBG 505 (by 195.9%) and RVSS 51 (by 260.0%) compared to JG 11. In the pot experiment, drought stress reduced biomass in the sensitive recurrent parents at the reproductive and pod-filling stages (Supplementary Table S7). While no significant differences were observed for root biomass, shoot biomass differed between the ILs and their recurrent and donor parents under the well-watered (WW) and water stress (WS) conditions. At the reproductive stage, DIBG 505 had significantly higher shoot fresh weight than its sensitive recurrent parent JG 11 and similar shoot fresh weight to its donor parent ICC 4958 under WS conditions. At the pod-filling stage under WS conditions, the parental genotypes of BGM 10216 differed, with the donor parent ICC 4958 having significantly higher shoot fresh weight and total fresh weight than the sensitive recurrent parent Pusa 372. BGM 10216 had significantly increased shoot fresh weight (by 41.6%) and total fresh weight (21.9%) compared to Pusa 372 (Supplementary Table S7).

### ‘*QTL-hotspot*’ enhances transpiration efficiency under water stress

**Fig. 3.**
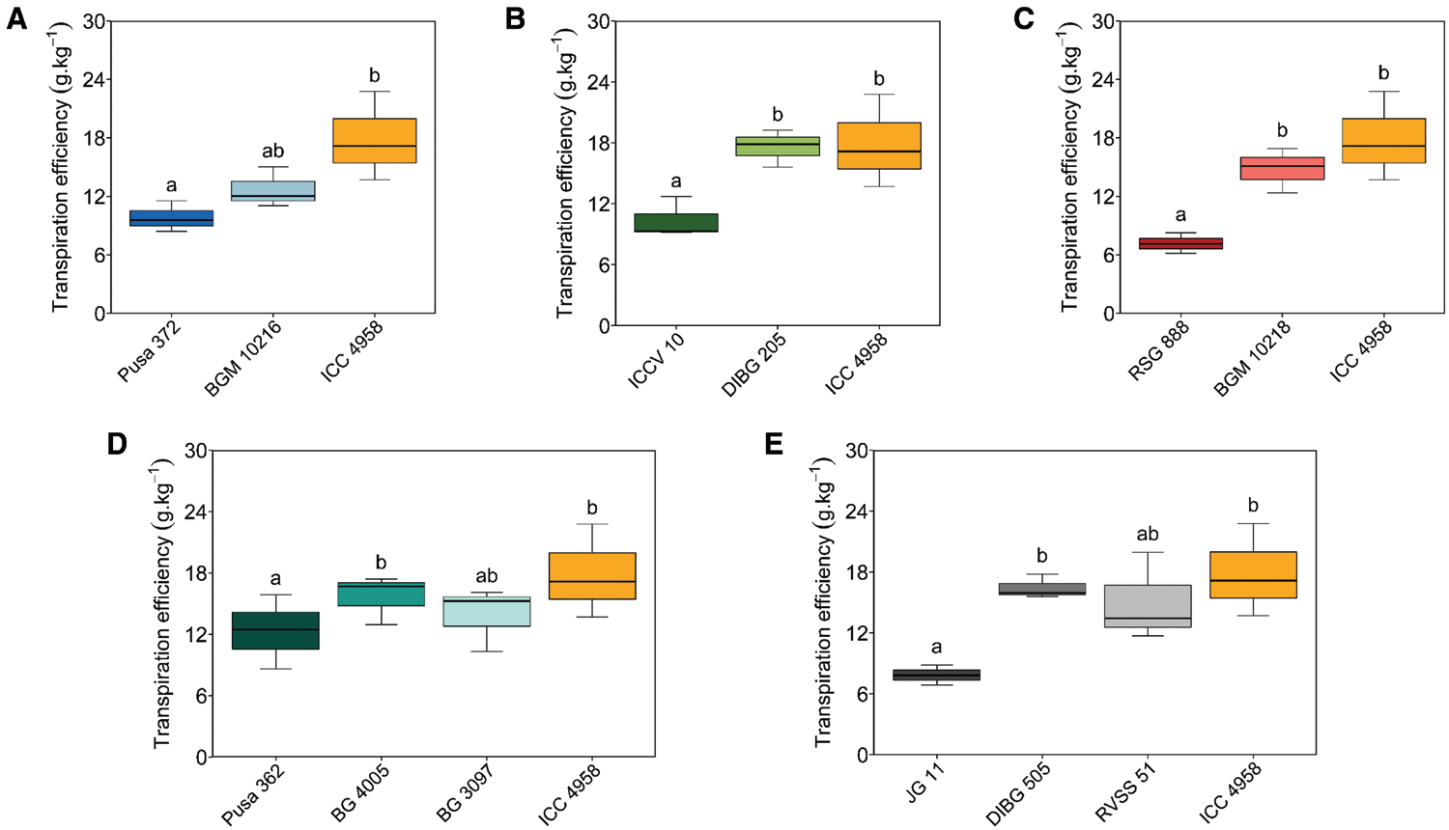
Effects of ‘*QTL-hotspot*’ on transpiration efficiency in chickpea introgression lines (ILs) and their parental lines under water deficit. Plants were grown in pots in a glasshouse at ICRISAT, Patancheru, India, and subjected to controlled water stress conditions from 28 d after sowing (see Methods). Details of the lines are given in Table 1. The first-named line within each graph is the recurrent parent, followed by the IL(s), and then the donor of ‘*QTL-hotspot*’, ICC 4958. Box-plots of the transpiration efficiencies of the ILs (A) BGM 10216, (B) DIBG 205, (C) BGM 10218, (D) BG 4005 and BG 3097, and (E) DIBG 505 and RVSS 51. The boxes denote the 25th and 75th percentiles, whiskers denote the full data range, and the center lines denote the medians. Different letters indicate significant differences among genotypes as determined using ANOVA followed by Tukey’s test (*P<*0.05).

The pattern of water uptake in the different genotypes was measured for 4 weeks after initiating drought stress at 28 DAS. BGM 10216 had significantly higher pre-anthesis water uptake than Pusa 372 under WW conditions, but not under WS conditions (Supplementary Table S8). No significant differences were observed in the water-use patterns for the ILs and their parental lines under WS conditions (Supplementary Fig. S4). The transpiration efficiency (TE) of the ILs at the pod-filling stage showed a relative improvement compared to their recurrent parents under water stress conditions (Supplementary Table S8). The parental genotypes differed under WS, with the drought-adapted parent ICC 4958 having a higher TE than the sensitive parents ICCV 10, RSG 888, and JG 11. Under WS conditions, DIBG 205, BGM 10218, and DIBG 505 had TEs similar to the donor parent (ICC 4958) and significantly higher than those of the recurrent parents ICCV 10 (80.0%), RSG 888 (114.3%), and JG 11 (100.0%), respectively (Fig. 3). Under WW conditions, DIBG 205 had significantly higher TE than the recurrent parent ICCV 10 (Supplementary Fig. S5). For all the other ILs except BGM 10218, TE did not significantly differ from their recurrent parental lines.

Carbon isotope discrimination (∆^13^C) did not differ significantly between the ILs and their recurrent parents under WW conditions (Supplementary Table S6). However, a small increase in ∆^13^C occurred for BG 4005 (2.9%) and BG 3097 (9.6%) compared to the recurrent parent Pusa 362. Similar results were found for BGM 10216 and DIBG 505 compared to the recurrent parents Pusa 372 and JG 11, respectively. For all the other ILs, ∆^13^C decreased relative to their recurrent parents.

### Relationships between yield parameters, phenology, canopy growth, and water extraction patterns

We next compared seed yield, yield components, and phenology traits under rainfed field conditions across each of the two mega-environments. In the first mega-environment (Gulbarga, Vijayapur, Badnapur, and Sehore), seed yield had a significant positive correlation with days to maturity (*R*=0.58, *P*<0.05) and 100-seed weight had a significant negative correlation with days to 50% flowering (*R*=–0.59, *P*<0.05). In the second mega-environment (Coimbatore, Arnej, Rahuri, and Nandyal), seed yield had significant negative correlations with days to 50% flowering (*R*=–0.72, *P*<0.01) and days to maturity (*R*=–0.79, *P*<0.01). In addition, 100-seed weight had significant negative correlations with days to 50% flowering (*R*=–0.86, *P*<0.001) and days to maturity (*R*=–0.70, *P*<0.05).

**Fig. 4.**
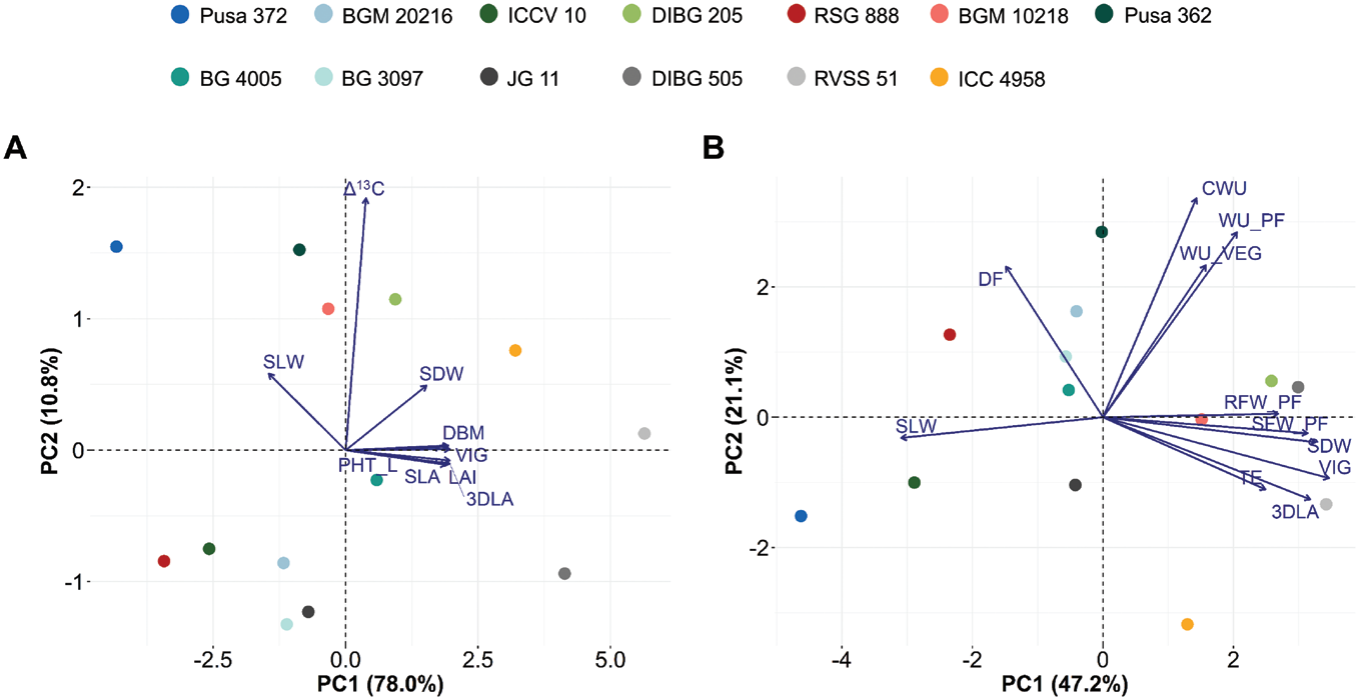
Principal component analysis (PCA) for phenotypic traits in chickpea introgression lines (ILs) and their parental lines evaluated under well-watered conditions. Plants were grown using the LeasyScan platform and in pots at ICRISAT, Patancheru, India. Details of the lines are given in Table 1. Principle components are shown for (A) canopy growth traits measured using the LeasyScan platform, and (B) traits related to water-use measured for plants grown in pots and canopy growth traits evaluated using the LeasyScan platform. The traits are as follows: 3DLA, 3D-leaf area; PLA, projected leaf area; DBM, digital biomass; SLW, specific leaf weight; SLA, specific leaf area; LAI, leaf area index; PHT_L, plant height; SDW, shoot dry weight; VIG, plant vigour; ∆^13^C, carbon isotope discrimination; DF, days to 50% flowering; TE, transpiration efficiency; SFW_PF, shoot fresh weight at pod-filling stage; RFW_PF, root fresh weight at pod-filling stage; WU_VEG, pre-anthesis water-use; WU_PF, post-anthesis water-use; and CWU, cumulative water-use.

PCA identified relationships between traits within and between experiments. Traits related to early plant vigour (3D-leaf area, projected leaf area, leaf area index, digital biomass, and vigour score) measured using the LeasyScan platform were closely linked (Fig. 4A). ∆^13^C was closely related to shoot dry weight and specific leaf weight. The PCA factor graph provided insights into the relationships between biomass and water-use traits measured in pot experiment. Under water deficit, increased pre-anthesis water-use was closely related to root biomass, shoot biomass, and TE measured at the pod-filling stage (Supplementary Fig. S6A). In addition, TE was closely associated with root and shoot biomass traits under WW conditions (Supplementary Fig. S6B). We compared canopy growth traits measured using the LeasyScan platform with traits related to water-use measured in pot experiment under WW conditions to identify the relationship between early vigour and water-use patterns (Fig. 4B). Shoot biomass evaluated by LeasyScan and in pot culture were closely correlated, highlighting the similarity between these two platforms. Notably, the PCA factor graph revealed a positive association between TE and 3D-leaf area.

### Whole-genome sequencing of ILs and parental lines reveals putative candidate genes underlying the ‘*QTL-hotspot*’ region

We performed whole-genome sequencing of the chickpea ILs and their parental lines to identify variations in chromosomes after the breeding process and to identify candidate genes underlying the ‘*QTL-hotspot*’ region. The sequencing data yielded ~0.49 billion clean reads after applying a filter on 0.65 billion raw reads, based on the Q30 score (Supplementary Table S9). Alignment of the clean reads with the chickpea draft genome assembly (Varshney *et al.* 2013b) provided ~9.03X average mapping depth and 98.63% genome coverage (Supplementary Table S9). A total of 941 432 SNPs were identified across 10 genotypes, of which 923 401 were biallelic (Supplementary Table S10). Considering the biallelic SNPs across eight pseudomolecules (Ca1 to Ca8), Ca4 had the most (187 443) and Ca8 had the least (19 796), accounting for 20.30% and 2.14% of the SNPs, respectively. We investigated the introgressed genomic regions in ILs derived from ICC 4958 in the ~300-kb interval of the ‘*QTL-hotspot*’ region delineated by the bin markers bin_4_13239546 and bin_4_13547009 (Kale *et al.*, 2015). On average, most SNPs were identified in the intergenic regions (82.02%), followed by intronic (8.99%) and exonic (8.99%) regions. Of the SNPs within the exonic regions, about 62.50% were missense, and 32.50% were silent mutations.

We selected polymorphic SNPs within 26 gene models underlying the ‘*QTL-hotspot*’ region common among the ILs to investigate potential candidate genes. Analysis of the ‘*QTL-hotspot*’ region in five ILs revealed that BGM 10216, DIBG 205, and BGM 10218 contained an introgressed region tagged by SNPs with the donor genotype (ICC 4958) (Supplementary Tables S11–S15) and shared five common SNPs in the coding or non-coding regions of four genes within the region. This included an intronic mutation (T598C) in *Ca_04557*, a 1-bp non-synonymous substitution in *Ca_04558* (A2922C), two synonymous SNP mutations in the exon of *Ca_04564* (G351C and C510T), and a SNP mutation in the intron of *Ca_04566* (T5632A). *Ca_04557* and *Ca_04558* were located within the ‘*QTL-hotspot_a*’ sub-region, while the ‘*QTL-hotspot_b*’ sub-region encompassed *Ca_04564* and *Ca_04566*. Gene ontology analysis based on the reference sequence of the kabuli chickpea cultivar CDC Frontier (Varshney *et al.*, 2013b) predicted that these four genes are involved in L-methionine biosynthesis, leaf development, protein binding, and phosphorylation processes (Supplementary Table S16).

Sequence analysis indicated that *Ca_04557* contains five exons and four introns, with an intronic mutation present within the second intron (Supplementary Fig. S7A). Based on homology with *Medicago truncatula*, *Ca_04557* is homologous with the *ARD1* gene encoding a 1,2 dihydroxy-3-keto-5-methylthiopentene dioxygenase 1 protein, regulating ethylene and polyamine biosynthesis (Sauter *et al.*, 2005). Therefore, we have designated *Ca_04557* as *CaARD1*. Analysis of sequence information revealed the presence of nine exons and eight introns within *Ca_04558*, with a non-synonymous mutation present within exon 6 (Supplementary Fig. S7B). A BLASTP analysis in the NCBI database (http://www.ncbi.nlm.nih.gov/BLAST) showed that the Ca_04558 protein is identical to BIG SEEDS1 (BS1), its homologous transcription factors in *M. truncatula* and *Glycine max* (Ge *et al.*, 2016). Hence, we have designated *Ca_04558* as *CaTIFY4b*. The alignment of the CaTIFY4b predicted protein sequence of the ILs, parental lines, and their homologs from different legume species revealed an amino acid substitution (I149S) within the highly conserved TIFY motif (Supplementary Fig. S8). Analysis of the *Ca_04564* sequence indicated the absence of introns (Supplementary Fig. S7C). *Ca_04564* is homologous to the *LRX2* gene in *M. truncatula*, encoding a LRR/extensin 2 protein, and hence we have designated it as *CaLRX2*. Finally, *Ca_04566* contained three exons and two introns, with an intronic mutation present within the second intron (Supplementary Fig. S7D). Based on homology with *M. truncatula*, *Ca_04566* is homologous to a *NET1A* gene encoding a NETWORKED 1A protein. Thus, we designated *Ca_04566* as *CaNET1A*.

### Validation of diagnostic KASP markers for drought adaptation

**Fig. 5.**
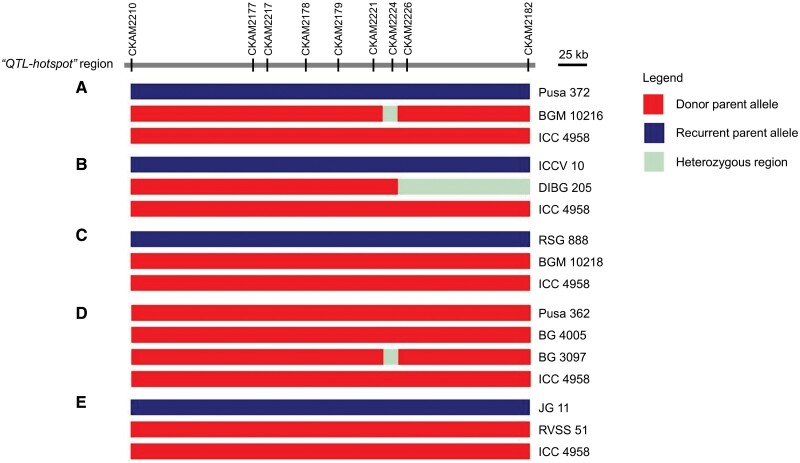
Validation of diagnostic markers for drought adaptation in chickpea. The KASP markers (designated as chickpea KASP assay markers, CKAMs) developed for nine single-nucleotide polymorphisms within the ‘*QTL-hotspot*’ region were validated in the introgression lines (ILs) (A) BGM 10216, (B) DIBG 205, (C) BGM 10218, (D) BG 4005 and BG 3097, and (E) RVSS 51. No polymorphisms were observed for the nine markers flanking the ~300-kb ‘*QTL-hotspot*’ region in Pusa 362, ICC 4958, and their ILs (BG 4005 and BG 3097). BGM 10216, DIBG 205, BGM 10218, and RVSS 51 had the expected graphical genotype.

KASP assays serve as a cost-effective fluorescence-based approach to genotype specific SNPs for low-density marker applications, such as trait screening and marker-assisted selection (Semagn *et al.*, 2014). We developed diagnostic KASP markers to validate the results obtained from the whole-genome sequencing analysis. Nine SNPs from the ~300-kb ‘*QTL-hotspot*’ region were selected to develop KASP genotyping assays (Supplementary Table S17), comprising four polymorphic SNPs prioritized based on whole-genome sequencing datasets, two SNPs each flanking the ‘*QTL-hotspot_a*’ and ‘*QTL-hotspot_b*’ sub-regions, and one SNP within the ‘*QTL-hotspot_b*’ sub-region. The diagnostic markers were verified using 12 genotypes, comprising six ILs, five recurrent parents, and one donor parent. All nine SNPs showed clear polymorphism between the recurrent and donor parents, except between Pusa 362 and ICC 4958 (Fig. 5). No polymorphism was observed between Pusa 362, ICC 4958, and the resultant ILs for the nine markers. Notably, the four KASP markers CKAM2177, CKAM2217, CKAM2221, and CKAM2226 developed for SNPs within *Ca_04557* (T598C), *Ca_04558* (A2922C), *Ca_04564* (G351C), and *Ca_04566* (T5632A), respectively, differentiated the parents and ILs (except those in the background of Pusa 362) for the presence/absence of ‘*QTL-hotspot*’ (Fig. 5). For instance, BGM 10216, DIBG 205, BGM 10218, and RVSS 51 carried drought-adaptive alleles from the donor parent (ICC 4958) for CKAM2177, CKAM2217, CKAM2221, and CKAM2226, except a heterozygous allele ‘A/T’ possessed by DIBG 205 for CKAM2226.

### The Chickpea Gene Expression Atlas provides insights into the expression patterns of the candidate genes

We examined the expression patterns of the four candidate genes underlying the ‘*QTL-hotspot*’ region using the *Cicer arietinum* Gene Expression Atlas, which includes RNA-seq datasets from 27 samples of ICC 4958 collected across five developmental stages (Kudapa *et al.*, 2018). Among the four genes, *CaARD1* had the high expression levels across tissues and developmental stages (Supplementary Fig. S9). *CaARD1* was highly expressed in leaves across all growth stages and in seeds at the senescence stage. In contrast, *CaLRX2* and *CaNET1A* had a low level of expression across all tissues and developmental stages. *CaLRX2* was expressed primarily in roots and nodules at the reproductive stage. The Gene Expression Atlas showed that *CaTIFY4b* was expressed in all tissues at the different developmental stages, with the highest expression in immature seeds at the reproductive stage. Since the ‘*QTL-hotspot*’ region improved seed weight and early vigour in the ILs, we hypothesized that *CaTIFY4b* is the candidate gene underpinning the causal mechanisms driving this QTL.

### Haplotype analysis of *CaTIFY4b* for 100-seed weight

**Fig. 6.**
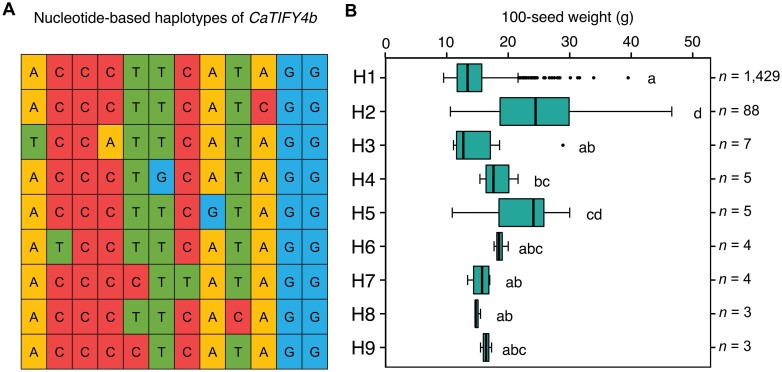
Haplotype analysis of the *CaTIFY4b* gene across 1548 desi accessions of the chickpea composite collection (Varshney *et al.,* 2021d). (A) Haplotypes of *CaTIFY4b* across the accessions. (B) Box-plot displaying the 100-seed weight distribution of each haplotype group within the collection. The boxes denote the 25th and 75th percentiles, whiskers denote the full data range, and the center lines denote the medians. Different letters indicate significant differences among the haplotype groups as determined using ANOVA followed by Duncan’s multiple range test. *n* indicates the number of accessions belonging to each group.

We used the sequencing and phenotyping data for 100-seed weight from 1548 desi accessions from Varshney *et al.* (2021d) to identify haplotypes for the *CaTIFY4b* gene, and a total of nine were detected (Fig. 6A). A significant variation in 100-seed weight was observed among them, and they were classified into different groups based on Duncan’s test (Fig. 6B). For instance, accessions with the *CaTIFY4b*-H2 haplotype possessed the highest 100-seed weight ranging up to 46.6 g; whereas, accessions containing the *CaTIFY4b*-H1 haplotype had the lowest 100-seed weight of 9.5 g. To further understand the superiority of the high-yielding donor parent ICC 4958 over the recurrent parents that we used, we identified and analysed the haplotype combinations for the *CaTIFY4b* gene. Interestingly, ICC 4958 possessed the superior haplotype *CaTIFY4b*-H2, while the recurrent parents Pusa 372, ICCV 10, and RSG 888 had *CaTIFY4b*-H1 for 100-seed weight. Sequence comparison of *CaTIFY4b*-H1 and *CaTIFY4b*-H2 revealed that these two haplotypes differed only at the non-synonymous SNP (A2922C; Fig. 6A), which affects the highly conserved TIFY motif of the CaTIFY4b protein, noted above. These results further supported our hypothesis that a non-synonymous mutation in *CaTIFY4b* might be associated with seed weight in chickpea. Further genetic and molecular characterization of the gene is needed to help extend our understanding of the control of organ size and drought adaptation mechanisms in chickpea.

## Discussion

Adaptation to drought stress in the later stages of development (terminal drought) has been a significant focus of chickpea crop improvement programs in Asia and sub-Saharan Africa for several decades (Varshney *et al.*, 2019). While several studies have increased our understanding of this complex trait in chickpea (Varshney *et al*., 2014, 2021a; Pang *et al.*, 2017; Roorkiwal *et al.*, 2020; Barmukh *et al.*, 2022), the genetic and physiological basis of drought adaptation remains largely unclear. In our present study, phenotypic and genotypic characterization of introgression lines (ILs) of ‘*QTL-hotspot*’ revealed that the drought adaptation mechanism of this region was associated with seed weight, phenology, canopy growth, and transpiration efficiency (TE). Understanding the impact of ‘*QTL-hotspot*’ on crop growth and development under terminal drought conditions provides insights into the causal mechanisms driving drought adaptation in chickpea.

To validate the role of ‘*QTL-hotspot*’ in improving seed yields under rainfed environments, we evaluated the ILs and parental lines through multi-location field trials in India. Under rainfed field conditions, the ILs BGM 10216, BGM 10218, BG 4005, and BG 3097 had significantly higher seed yields than their recurrent parent in at least three locations, while DIBG 505, RVSS 51, and DIBG 205 either had similar or decreased seed yields in all the tested locations (Fig. 1; Supplementary Table S2). Notably, BGM 10216 had a 21.4% higher pooled mean seed yield than the recurrent parent Pusa 372 and was less sensitive to changing environments. As a result, BGM 10216 has been released by the ICAR-AICRP on Chickpea as ‘Pusa Chickpea 10216’ for commercial cultivation in the central and south zones of India (Roorkiwal *et al.*, 2020; Bharadwaj *et al.*, 2021). In the present study, soil moisture content during the pod-filling stage distinguished two mega-environments for seed yield under drought conditions (Supplementary Fig. S2). These results are in accordance with previous studies on chickpea (Zaman-Allah *et al.*, 2011; Vadez *et al.*, 2012; Korbu *et al.*, 2022), which suggest that water availability during the pod-filling period is crucial for improving seed yield under water deficit. Our results revealed that the first mega-environment experienced mild drought stress during the pod-filling stage (Supplementary Table S4); hence, seed yield was found to be positively correlated with days to maturity in this mega-environment. By contrast, drought stress during the pod-filling stage was severe in the second mega-environment, and this was also reflected in a significant negative correlation observed between seed yield and time to flowering/maturity. A significant increase in seed yield was observed for most of the ILs over their recurrent parent across both mega-environments, and particularly in the second one (Fig. 1), and this highlights the importance of the ‘*QTL-hotspot*’ region in enhancing drought adaptation in chickpea. Furthermore, most of the ILs had higher 100-seed weights than their recurrent parents within a location and in the pooled mean across locations (Table 2). The positive correlation that we found between yield and 100-seed weight indicated that the ICC 4958 allele of ‘*QTL-hotspot*’ was a critical factor driving seed yield under drought conditions, which is in accordance with our previous study (Barmukh *et al.*, 2022). The selection of early-maturing genotypes with no significant yield penalty is of particular interest to plant breeders tackling terminal drought stress. Notably, the introgression of ‘*QTL-hotspot*’ significantly reduced days to 50% flowering and days to maturity in ILs compared to their recurrent parent, suggesting that ‘*QTL-hotspot*’ ILs avoided post-anthesis drought stress by completing their life cycle early, hence increasing yield in water-limited environments.

Previous studies in sorghum (Borrell *et al*., 2014a, 2014b) and pearl millet (Kholová *et al.*, 2009) showed that drought adaptation QTL(s) reduced canopy size at anthesis, decreased transpiration rate, and increased water extraction during the grain-filling stage, resulting in higher grain yield under drought conditions in the near-isogenic lines compared to their recurrent parents. In our present study, the ICC 4958 allele of ‘*QTL-hotspot*’ constitutively enhanced traits related to early vigour- such as leaf area, leaf area index, plant biomass, and plant height (Supplementary Table S6). Under terminal drought stress, even a small increase in TE can significantly improve legume seed yield (Vadez and Ratnakumar, 2016). Importantly, the ILs had higher TE than their recurrent parents under water deficit, and this was associated with the presence or absence of the ‘*QTL-hotspot*’ region (Fig. 3). Our results suggested that an increase in plant vigour (canopy growth) and TE, at least in part, contributed to an increase in seed yield under drought conditions. The most reasonable explanation for our results would be that an increase in leaf area due to the ICC 4958 allele of ‘*QTL-hotspot*’ is translated into enhanced canopy biomass and seed weight via the source–sink relationship. Our analysis supports the hypothesis of a causal link between leaf area and seed weight through the balance of carbohydrate supply and demand controlled by the ‘*QTL-hotspot*’ region. Furthermore, the results for ∆^13^C did not significantly differ between the ILs and their recurrent parents (Supplementary Table S6) and were not closely associated with plant vigour or canopy growth traits (Fig. 4). Previous studies of chickpea that have measured ∆^13^C and TE have reported the absence of an expected correlation under WW conditions (Kashiwagi *et al.*, 2006b; Turner *et al.*, 2007), suggesting that variation in ∆^13^C in chickpea is a consequence of stomatal responsiveness to changes in water demand. Overall, this suggests that the ‘*QTL-hotspot*’ region increases water-use by increasing vigour and traits related to canopy growth, rather than water-use efficiency at the leaf level.

Our whole-genome sequencing data provided insights into changes in the ‘*QTL-hotspot*’ region due to the breeding process. No polymorphism for target SNPs within the ‘*QTL-hotspot*’ region was observed between the ILs BG4005 and BG3097 and their parental lines Pusa 362 and ICC 4958. This was in accordance with the phenotypic data for BG 4005 and BG3097, which revealed no significant variation in the pooled means for 100-seed weight, traits related to early vigour, and TE over the recurrent parent Pusa 362 (Table 2; Supplementary Table S6, Supplementary Fig. S5). This reaffirmed the role of the ~300-kb interval of the ‘*QTL-hotspot*’ region for controlling multiple drought-adaptive traits including plant vigour in chickpea (Kale *et al.*, 2015; Sivasakthi *et al.*, 2018). The polymorphic SNPs within the ‘*QTL-hotspot*’ region identified among the parental lines and their corresponding ILs facilitated the identification of four genes that represented putative candidates for the different drought-adaptive traits. In addition to the candidate genes *CaARD1* and *CaTIFY4b* that we identified in our previous study (Barmukh *et al.*, 2022), here we also identified *CaLRX2* and *CaNET1A* as the potential genes underlying the ‘*QTL-hotspot*’ region (Supplementary Fig. S7). This is in accordance with the fact that drought adaptation is a complex trait that may be regulated by multiple genes (Varshney *et al.*, 2021a). Several homologs of the candidate genes have previously been reported to be associated with drought and/or salinity stress tolerance in plants. For instance, *ARD1* belongs to a metal-binding protein family (acireductone dioxygenase), and its homologous gene in rice is *OsARD1*. In a recent study, overexpression of *OsARD1* was shown to improve tolerance to submergence, drought, and salinity stress in rice by enhancing ethylene synthesis (Liang *et al.*, 2019). The leucine-rich repeat extensin proteins LRX 3/4/5 were found to function together with RALF22/23 and FER to regulate plant growth and salt tolerance in Arabidopsis (Zhao *et al.*, 2018). In addition, *NET1A* belongs to the Networked superfamily of actin-binding proteins, which specify diverse membrane compartments in plant cells and promote actin–membrane interactions (Deeks *et al.*, 2012). *NET1A* in cotton is induced in response to drought stress (Li *et al.*, 2019). Further studies are required to validate the contribution of each candidate gene underlying the ‘*QTL-hotspot*’ region to the observed phenotype.

Based on our present study, we hypothesize that a non-synonymous variation in the plant-specific transcription factor TIFY4b might be responsible for improving the drought adaptation of ‘*QTL-hotspot*’ ILs. This is supported by haplotype analysis of *CaTIFY4b* for 100-seed weight across 1548 desi accessions of a chickpea composite collection, where the donor parent ICC 4958 was found to possess the superior haplotype *CaTIFY4b*-H2 that differed from *CaTIFY4b*-H1 by a non-synonymous SNP (A2922C; Fig. 6). This might be one of the major reasons for its superior performance in terms of seed weight. These results are in line with our previous study (Barmukh *et al.*, 2022), which showed that a genetic variation in *CaTIFY4b* improves seed weight, organ size, and drought adaptation in chickpea. *TIFY4b* encodes a type-II TIFY transcription regulator (Vanholme *et al.*, 2007; Bai *et al.*, 2011). It is homologous to *BIG SEEDS1* (*BS1*) in *Medicago* and soybean that regulates seed weight and organ size (Ge *et al.*, 2016). It is also related to two Arabidopsis genes, *PEAPOD 1* (*PPD1*) and *PPD2*, which regulate leaf size (White, 2006). The highly conserved TIFY motif mediates homo- and heteromeric interactions between JASMONATE ZIM-domain (JAZ) proteins in Arabidopsis (Chung *et al.*, 2009). Therefore, we predict that the presence of a non-synonymous substitution in the TIFY motif of *CaTIFY4b* affects protein–protein interactions. *CaTIFY4b* is a strong candidate for ‘*QTL-hotspot*’ effects due to the close correspondence observed between traits associated with the ILs and the phenotype of *CaTIFY4b* homologs in different legume species.

In summary, our study offers insights into the ‘*QTL-hotspot*’ region that contains adaptive alleles for the key abiotic stress of drought. The candidate genes and validated diagnostic markers underpinning the ‘*QTL-hotspot*’ region identified in this study will be significant for developing drought-adaptive varieties of chickpea for the future.

## Supplementary data

The following supplementary data are available at *JXB* online.

Fig. S1. Marker-assisted backcrossing scheme used to develop ‘*QTL-hotspot*’ introgression lines in five elite genetic backgrounds using ICC 4958 as the donor.

Fig. S2. Bi-plots of genotype and genotype-by-environment interactions for genotypes evaluated under rainfed conditions at the multi-location field trials.

Fig. S3. Time-courses of 3D-leaf area in the introgression lines and their recurrent parents evaluated using the LeasyScan platform under well-watered conditions.

Fig. S4. Time-courses of water-use profile in the introgression lines and their parental lines grown in pots under water stress conditions.

Fig. S5. Effect of ‘*QTL-hotspot*’ on transpiration efficiency in the introgression lines and their parental lines grown in pots under well-watered conditions.

Fig. S6. Principal component analysis for phenotypic traits evaluated in the introgression lines and their parental lines grown in pots under water stress and well-watered conditions.

Fig. S7. Structure of the candidate genes underlying the ‘*QTL-hotspot*’ region.

Fig. S8. Comparison of identified amino acid sequences between *CaTIFY4b* and its homologs in other legume plants.

Fig. S9. *In silico* analysis of *CaARD1*, *CaTIFY4b*, *CaLRX2*, and *CaNET1A* expression in different tissues at different development stages.

Table S1. Summary of traits evaluated in the field, LeasyScan, and pot experiments.

Table S2. Descriptive statistics of traits evaluated in the field, LeasyScan, and pot experiments.

Table S3. ANOVA results for seven introgression lines and their parental lines evaluated in the field and pot experiments.

Table S4. Soil moisture content recorded at 30-d intervals during the growing season at the different field locations.

Table S5. Total rainfall measured over 30-d intervals during the growing season at the different field locations.

Table S6. Canopy growth and biomass traits of the introgression lines and their parental lines evaluated in the LeasyScan experiment.

Table S7. Distribution of biomass between roots and shoots in the introgression lines and their parental lines at the reproductive and pod-filling stages under well-watered and water stress conditions in the pot experiment.

Table S8. Water-use at different development stages for the introgression lines and their parental lines under well-watered and water stress conditions in the pot experiment.

Table S9. Summary of whole-genome sequencing reads generated and the alignment statistics of the introgression lines and their parental lines.

Table S10. Summary of raw, filtered, and biallelic SNPs in the introgression lines and their parental lines.

Table S11. Polymorphic SNPs underlying the *‘QTL-hotspot’* region found in BGM 10216 and its parental lines.

Table S12. Polymorphic SNPs underlying the *‘QTL-hotspot’* region found in DIBG 205 and its parental lines.

Table S13. Polymorphic SNPs underlying the *‘QTL-hotspot’* region found in BGM 10218 and its parental lines.

Table S14. Polymorphic SNPs underlying the *‘QTL-hotspot’* region found in BG 4005 and its parental lines.

Table S15. Polymorphic SNPs underlying the *‘QTL-hotspot’* region found in BG 3097 and its parental lines.

Table S16. List of candidate genes within the *‘QTL-hotspot’* region based on the reference sequence of the kabuli chickpea cultivar CDC Frontier.

Table S17. Primers used for the development of KASP markers within the *‘QTL-hotspot’* region.

erac348_suppl_Supplementary_Figures

erac348_suppl_Supplementary_Tables

## Data Availability

The whole-genome sequencing data reported in this study have been deposited in the NCBI SRA (https://www.ncbi.nlm.nih.gov/sra) with the BioProject ID PRJNA772517. The phenotyping data supporting this study’s findings have been deposited in figshare and are available at https://doi.org/10.6084/m9.figshare.16830709.

## Abbreviations

∆^13^C: Carbon isotope discrimination
CKAM: chickpea KASP assay marker
DAS: days after sowing
IL: introgression line
KASP: kompetitive allele specific PCR
MABC: marker-assisted backcrossing
PCA: principal component analysis
QTL: quantitative trait locus
SNP: single-nucleotide polymorphism
TE: transpiration efficiency
WS: water stress
WW: well-watered
