# Supplementary material for: Characterization of ‘QTL-hotspot’ introgression lines reveals physiological mechanisms and candidate genes associated with drought adaptation in chickpea
Source: J Exp Bot. 2022 Aug 25;73(22):7255–72. doi: 10.1093/jxb/erac348 (PMC9730794; doi:10.1093/jxb/erac348)
Supplement: erac348_suppl_Supplementary_Figures [file erac348_suppl_supplementary_figures.pdf]

Characterization of *"QTL-hotspot"* introgression lines reveals physiological mechanisms and candidate genes associated with drought adaptation in chickpea

Rutwik Barmukh[1,2], Manish Roorkiwal[1,3,#,*], Girish P. Dixit[4], Prasad Bajaj[1], Jana Kholova[5,6], Millicent R. Smith[1,7], Annapurna Chitikineni[1], Chellapilla Bharadwaj[3,8], MS Sheshshayee[9], Abhishek Rathore[1], Shailesh Tripathi[8], Mohammad Yasin[10], Adiveppa G. Vijayakumar[11], Someswar Rao Sagurthi[2], Kadambot H.M. Siddique[3], Rajeev K. Varshney[1,3,12,*]

[1]Centre of Excellence in Genomics and Systems Biology, International Crops Research Institute for the Semi-Arid Tropics (ICRISAT), Hyderabad, India

[2]Department of Genetics, Osmania University, Hyderabad, India

[3]The UWA Institute of Agriculture, The University of Western Australia, Perth, Western Australia, Australia

[4]ICAR - Indian Institute of Pulses Research (IIPR), Kanpur, India

[5]Crops Physiology & Modeling, International Crops Research Institute for the Semi-Arid Tropics (ICRISAT), Hyderabad, India

[6]Department of Information Technologies, Faculty of Economics and Management, Czech University of Life Sciences Prague, Kamýcká 129, Prague, Czech Republic

[7]Queensland Alliance for Agriculture and Food Innovation, The University of Queensland, Australia

[8]ICAR - Indian Agricultural Research Institute (IARI), Delhi, India

[9]Department of Crop Physiology, University of Agricultural Sciences, Bengaluru, India

[10]RAK College of Agriculture, Rajmata Vijayaraje Scindia Krishi Vishwa Vidyalaya, Gwalior, India

[11]UAS-Dharwad Regional Agricultural Research Station, Vijayapura, India

[12]State Agricultural Biotechnology Centre, Centre for Crop and Food Innovation, Murdoch University, Murdoch, Western Australia, Australia

[#]Present address: Khalifa Center for Genetic Engineering and Biotechnology, United Arab Emirates University, Al-Ain, United Arab Emirates

* Correspondence: mroorkiwal@uaeu.ac.ae (Manish Roorkiwal);
rajeev.varshney@murdoch.edu.au (Rajeev K. Varshney)

Supplementary Figures:

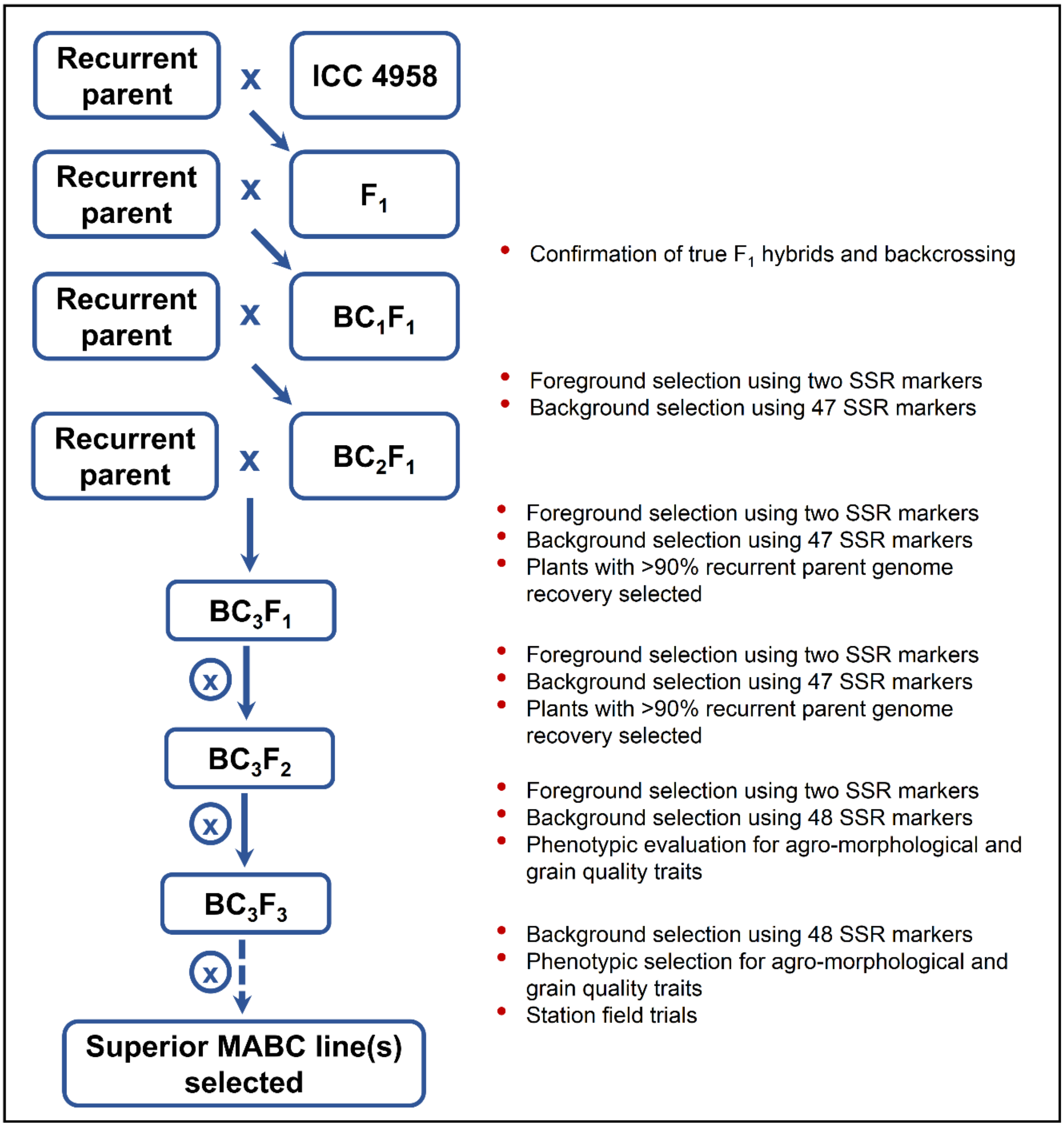

Supplementary Fig. S1: Marker-assisted backcrossing (MABC) scheme to develop "*QTL-hotspot*" ILs with enhanced drought adaptation in five elite genetic backgrounds of chickpea using ICC 4958 as the donor. NCPGR21 and NCPGR127 markers were used for foreground selection.

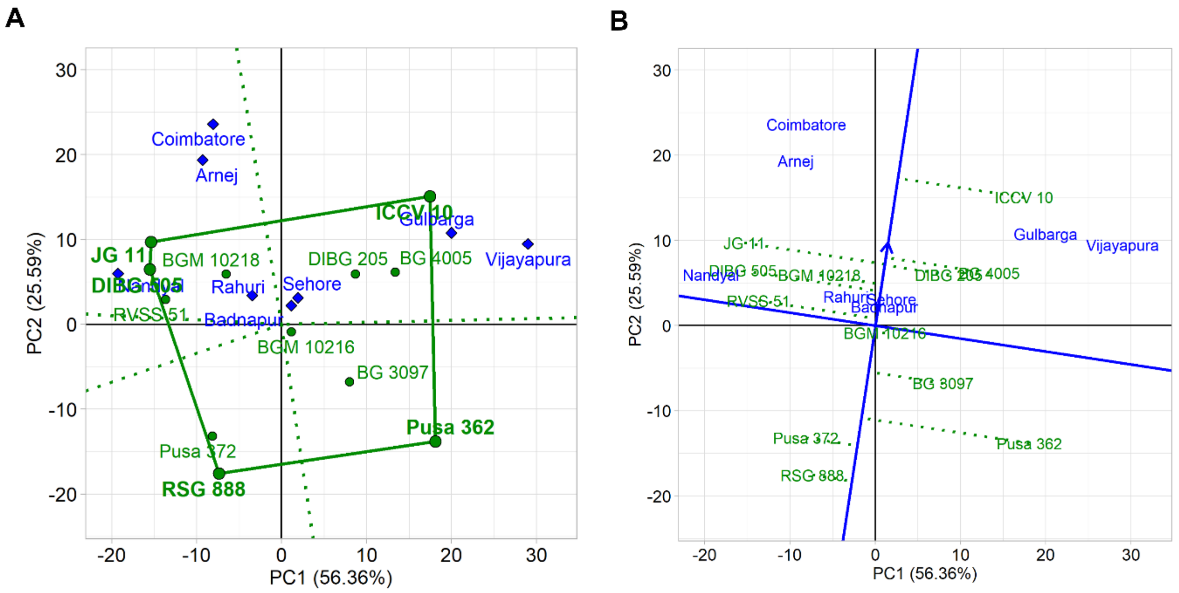

Supplementary Fig. S2: Genotype and genotype-by-environment interaction (GGE)-biplot analysis for chickpea genotypes evaluated under multi-location field trials. (A) '*Which-won-where*' view of GGE biplot for chickpea ILs and their recurrent parents contrasting for yield under rainfed field conditions. Green circles indicate different genotypes and blue diamonds indicate diverse locations for genotype evaluation. (B) Average-environment coordination (AEC) view depicting the mean yield performance and stability of chickpea ILs and their recurrent parents based on GGE biplot analysis.

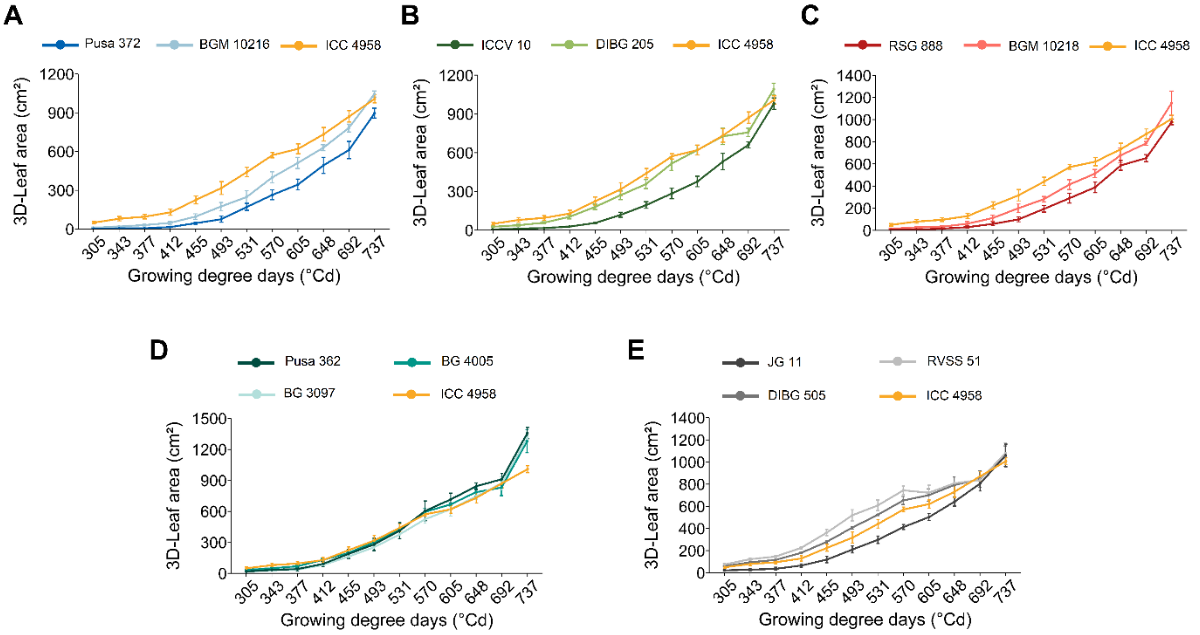

Supplementary Fig. S3: Time course of 3D-leaf area in ILs and their respective recurrent parent evaluated at LeasyScan platform. Time course of 3D-leaf area in (A) Pusa 372, BGM 10216, and ICC 4958; (B) ICCV 10, DIBG 205, and ICC 4958; (C) RSG 888, BGM 10218, and ICC 4958; (D) Pusa 362, BG 4005, BG 3097, and ICC 4958; (E) JG11, DIBG 505, RVSS 51, and ICC 4958. The ILs along with their recurrent parent were evaluated at LeasyScan platform during vegetative growth under well-watered conditions. Data are mean ± S.E. (*n*=3).

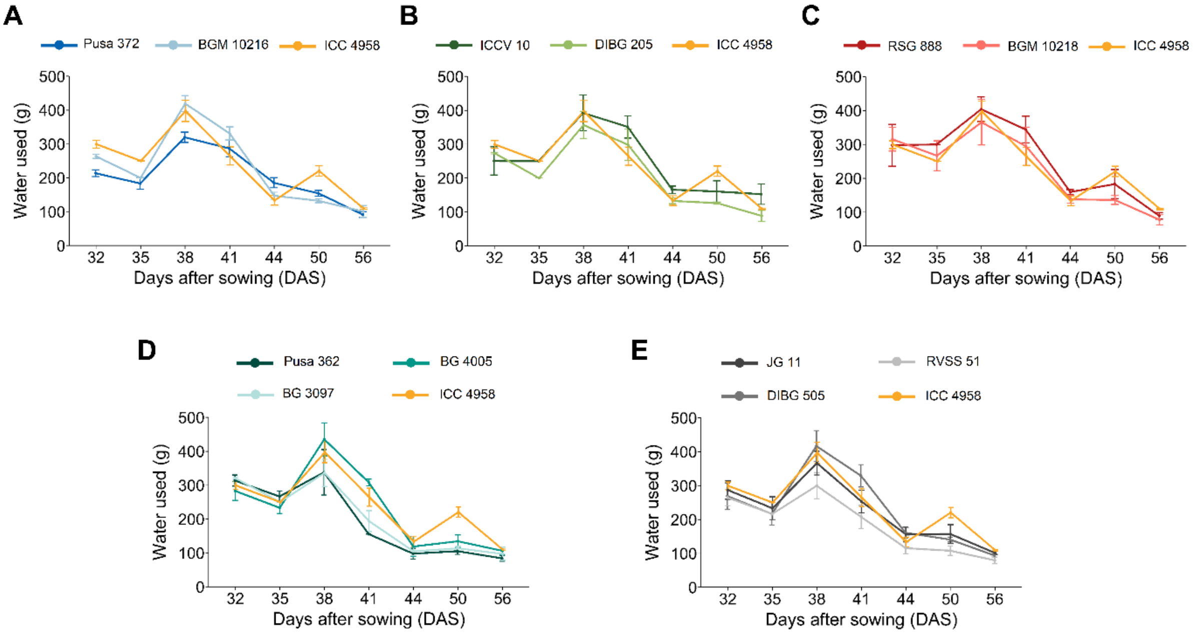

Supplementary Fig. S4: Variation in water use profile in ILs and their parental lines grown under water stress conditions. Time course of water uptake in (A) Pusa 372, BGM 10216, and ICC 4958; (B) ICCV 10, DIBG 205, and ICC 4958; (C) RSG 888, BGM 10218, and ICC 4958; (D) Pusa 362, BG 4005, BG 3097, and ICC 4958; (E) JG11, DIBG 505, RVSS 51, and ICC 4958. Plants were grown in pots under drought stress conditions. Data are mean ± S.E. (*n*=3).

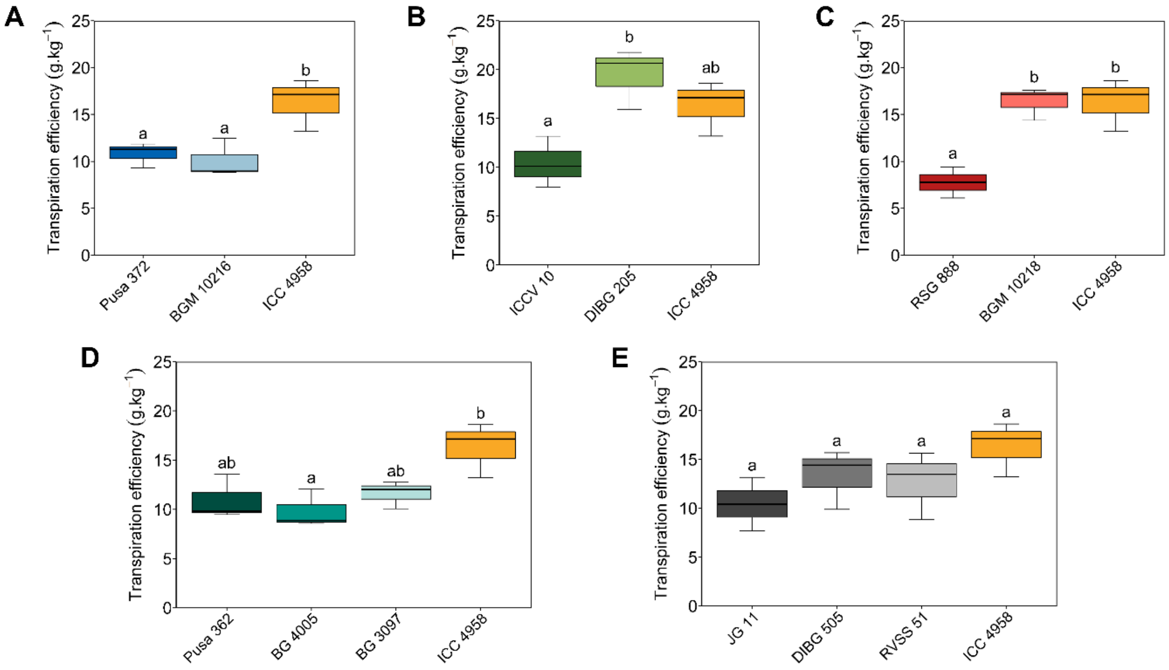

Supplementary Fig. S5: Effect of *"QTL-hotspot"* on transpiration efficiency measured in pots under well-watered conditions. Comparison of transpiration efficiency in parental lines and ILs: (A) BGM 10216, (B) DIBG 205, (C) BGM 10218, (D) BG 4005 and BG 3097, and (E) DIBG 505 and RVSS 51. For the box plots, boxes denote the 25th and 75th percentiles, whiskers denote the full data range, and center lines denote medians. Different letters above the boxes indicate statistical significance between genotypes using Tukey's test ($P < 0.05$).

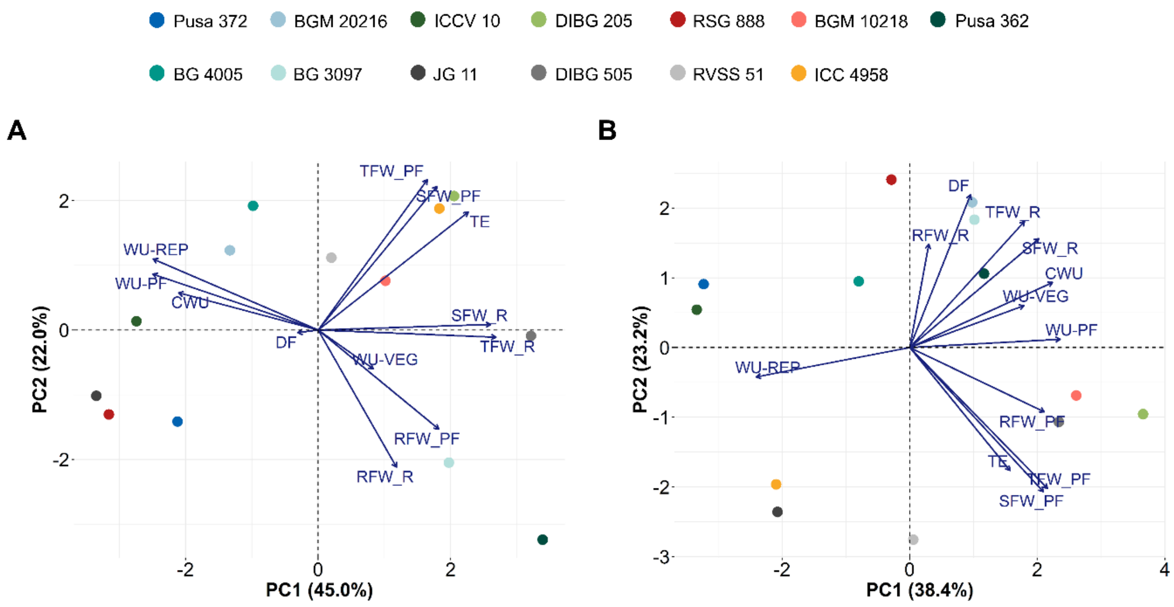

Supplementary Fig. S6: Principal component analysis for phenotypic traits evaluated using pot culture. (A,B) Principal component analysis (PCA) factor graphs for ILs and their respective recurrent parents evaluated under (A) water stress and (B) well-watered conditions. Traits are indicated by their respective trait codes (see Supplementary Table S2). In the PCA, positively correlated variables are placed together, while negatively correlated variables are on opposite sides of the origin. The individual contribution of each trait to each dimension (in percent) is denoted in the axis legend. The positions of the colored circles correspond to specific trait loadings with respect to principal component 1 (PC1) and principal component 2 (PC2).

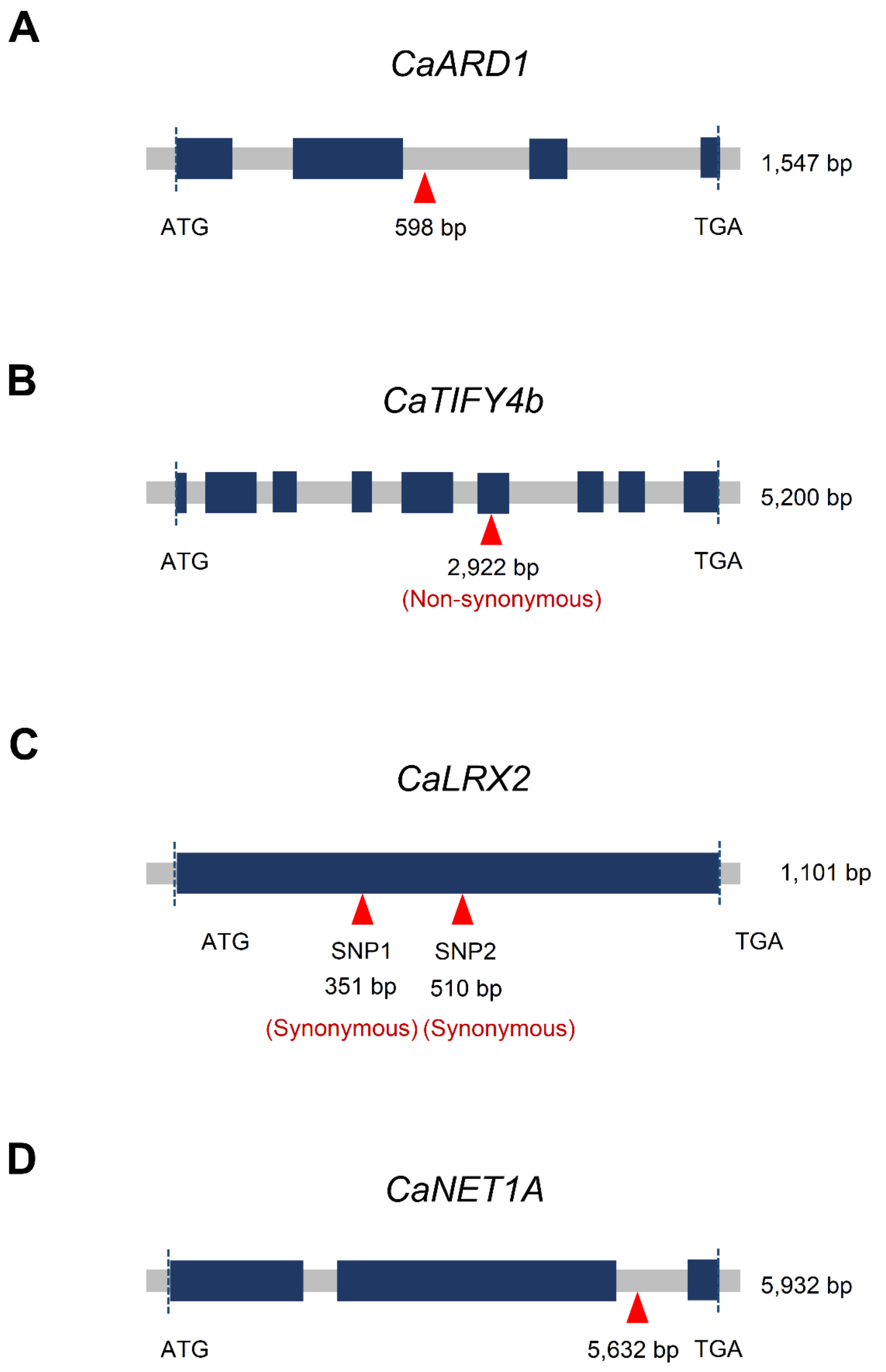

Supplementary Fig. S7: Structure of the prioritized genes underlying the *"QTL-hotspot"* region. Structure of (A) *CaARD1* gene based on annotation of *Ca_04557*, (B) *CaTIFY4b* gene based on annotation of *Ca_04558*, (C) *CaLRX2* gene based on annotation of *Ca_04564*, and (D) *CaNET1A* gene based on annotation of *Ca_04566*. Blue rectangles, open-reading frame (ORF); red arrowhead, single nucleotide substitution in introgression line relative to the recurrent parent.

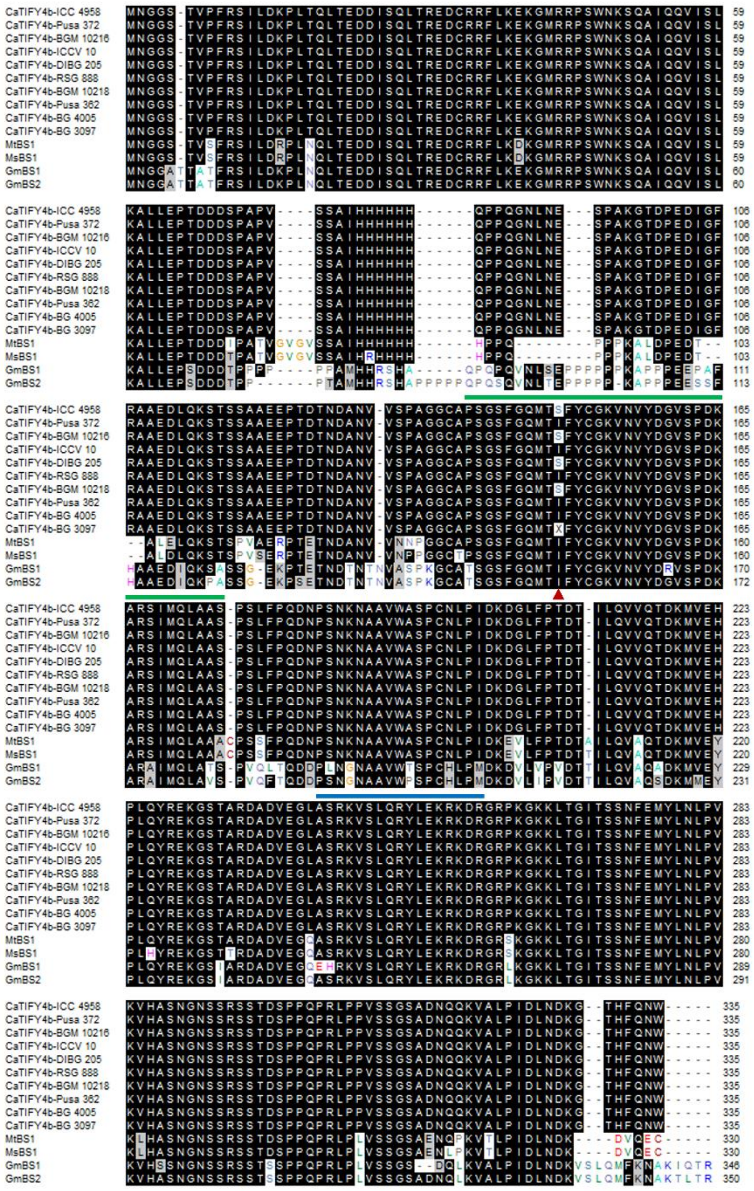

Supplementary Fig. S8: Comparison of identified amino acid sequences between *CaTIFY4b* and its homologs in other legume plants. CaTIFY4b-ICC 4958, CaTIFY4b of chickpea cv. ICC 4958; CaTIFY4b-Pusa 372, CaTIFY4b of chickpea cv. Pusa 372; CaTIFY4b-BGM 10216, CaTIFY4b of chickpea introgression line BGM 10216; CaTIFY4b-ICCV 10, CaTIFY4b of chickpea cv. ICCV 10; CaTIFY4b-DIBG 205, CaTIFY4b of chickpea introgression line DIBG 205; CaTIFY4b-RSG 888, CaTIFY4b of chickpea cv. RSG 888; CaTIFY4b-BGM 10218, CaTIFY4b of chickpea introgression line BGM 10218; CaTIFY4b-Pusa 362, CaTIFY4b of chickpea cv. Pusa 362; CaTIFY4b-BG 4005, CaTIFY4b of chickpea introgression line BG 4005; CaTIFY4b-BG 3097, CaTIFY4b of chickpea introgression line BG 3097. Non-chickpea sequences are MtBS1 (*Medicago truncatula*; KM668032), MsBS1 (*Medicago sativa*; KM668033), GmBS1 (*Glycine max*; KM668027), GmBS2 (*Glycine max*; KM668028). Identical amino acid residues across accessions are shaded black. Red arrowhead at the bottom of the sequence indicates the location of a non-synonymous point mutation in ICC 4958. Green and blue bars above the sequence indicate TIFY domain, and putative TIFY and CCT2 domains, respectively, as predicted by InterPro (http://www.ebi.ac.uk/interpro/).

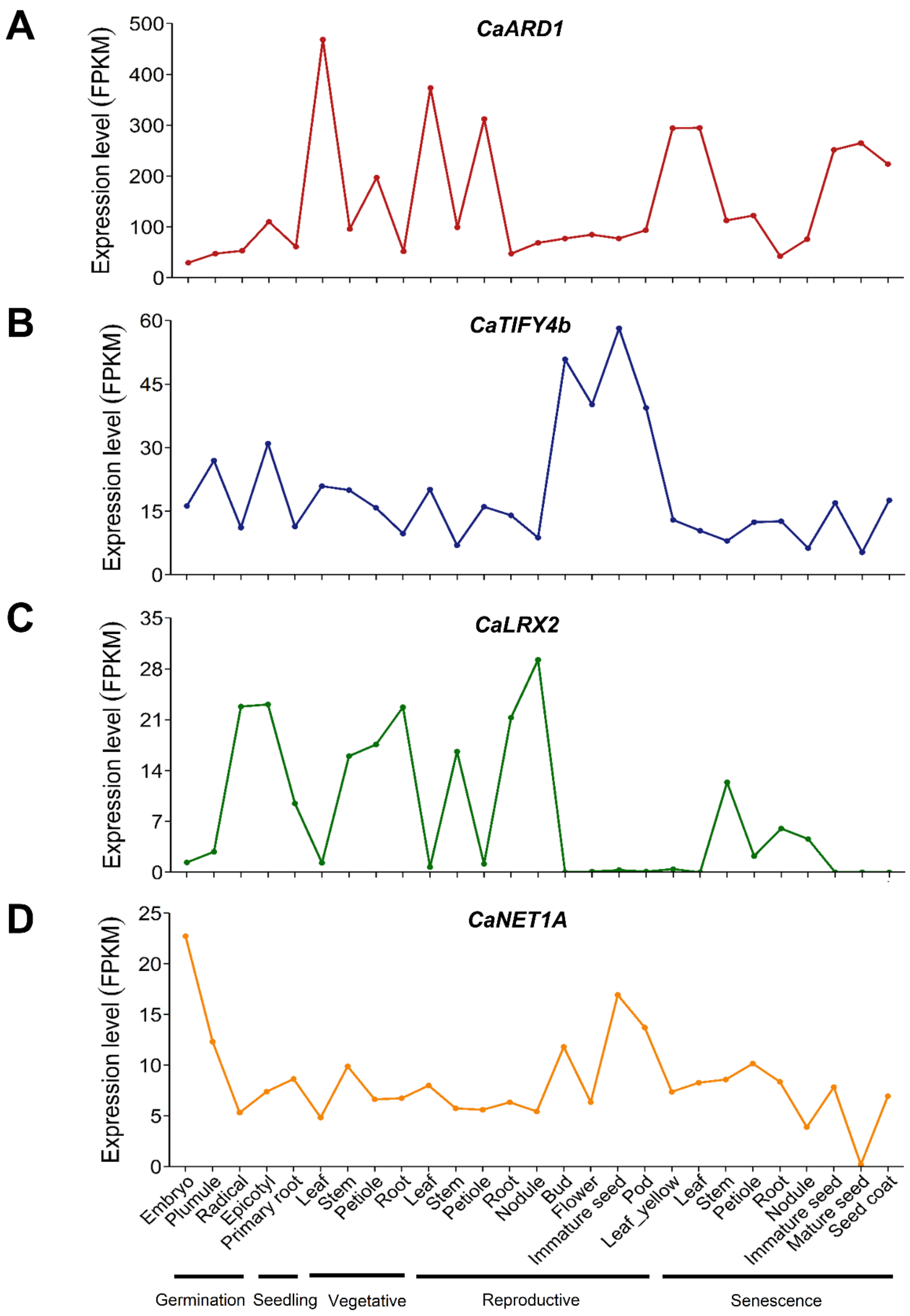

Supplementary Fig. S9: *In silico* analysis of *CaARD1*, *CaTIFY4b*, *CaLRX2*, and *CaNET1A* gene expression. Expression of (A) *CaARD1*, (B) *CaTIFY4b*, (C) *CaLRX2*, and (D) *CaNET1A* gene expression in different tissues as shown by *Cicer arietinum* Gene Expression Atlas.
